# Continuous flow photocyclization of stilbenes – scalable synthesis of functionalized phenanthrenes and helicenes

**DOI:** 10.3762/bjoc.9.221

**Published:** 2013-09-17

**Authors:** Quentin Lefebvre, Marc Jentsch, Magnus Rueping

**Affiliations:** 1 Institute of Organic Chemistry, RWTH Aachen University, Landoltweg 1, D-52074 Aachen, Germany

**Keywords:** continuous-flow reactor, flow chemistry, helicenes, light-driven cyclization reaction, photocyclization, stilbenes

## Abstract

A continuous flow oxidative photocyclization of stilbene derivatives has been developed which allows the scalable synthesis of backbone functionalized phenanthrenes and helicenes of various sizes in good yields.

## Introduction

Phenanthrenes are versatile intermediates toward polycyclic aromatic hydrocarbons which are relevant for materials sciences, as well as toward helicenes, an intriguing class of molecules which show remarkable chiroptical properties due to their helical pitch. The rapidly expanding field of application of helicene-like molecules in materials sciences and optics demands the development of scalable and flexible syntheses [1–5].

Following the pioneering examples of Scholz [6] and Martin [7] in 1967, the photocyclization of stilbene derivatives under UV-light irradiation is now a classical method for the synthesis of phenanthrenes and helicene-like molecules [8]. However, the scalability of these reactions is limited by the required low concentration (~10^−3^ mol·L^−1^) and usually long reaction times (>20 h). Therefore, most of the applications of this method are limited to small scale (<0.5 mmol), making it unsuitable for gram-scale synthesis of helicene-like molecules [9–12]. Much effort has devoted to the development of alternative pathways toward helicenes, but most approaches require lengthy syntheses of the precursors [13–15]. It should be noted that an elegant semi-one-pot procedure for the synthesis of phenanthrenes from styrenes and benzene was recently reported [16]. However, also in this case, the scale of the reaction is limited by the size of the photoreactor.

**Scheme 1:**
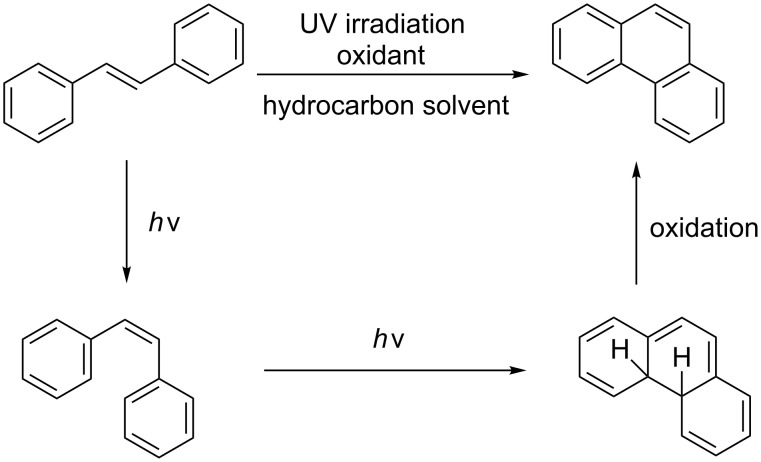
Photocyclization of stilbene to phenanthrene.

Reactions in flow are typically faster and cleaner compared to the corresponding reactions in batch. A flow setup is particularly well-suited for photo-catalysed reactions as the efficiency of the transformation is no longer related to the scale [17–21]. Therefore, the development of an efficient protocol for the photocyclization of stilbene derivatives in flow would be of great interest. A recent contribution described the flow-synthesis of [5]helicene under visible light in the presence of a sensitizer [22], but, to the best of our knowledge, no reports on broadly applicable light-induced oxidative photocyclizations in flow are known. Herein, we report the first photocyclization of polysubstituted olefins using a continuous flow process and discuss advantages and limitations of this new protocol. Generally, the oxidative photodehydrogenation of *E*-stilbene results in the formation of phenanthrene (Scheme 1). In this reaction the *E*-olefin (or a *E*/*Z* mixture of the olefin) is photoisomerised to the reactive *Z*-olefin, which undergoes photocyclization. The corresponding dihydrophenanthene is subsequently oxidized with iodine to give the desired phenanthrene and HI, which can be quenched by propylene oxide or THF.

## Results and Discussion

In order to accomplish a first general photocyclization we started with the photocyclization of stilbene. Optimization of the reaction conditions in a small flow setup (5 mL FEP tubing, 150 W UV-lamp) is presented in Table 1. Although both THF and propylene oxide showed good ability to quench HI, we choose THF as additive due to its lower cost, volatility and toxicity [23–24]. Under the optimized conditions, *E*-stilbene (**1a**) could be converted to phenanthrene (**2a**) in 95% NMR yield with a retention time of 83 min (Table 1, entry 5).

**Table 1:** Proof of principle and screening of reaction conditions.

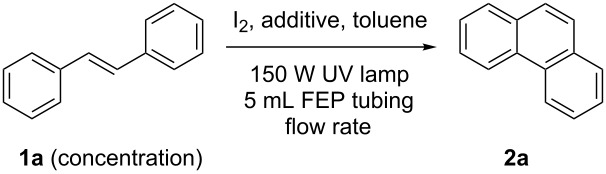				

Entry^a^	Additive (20 equiv)	Concentration (mol/L)	Flow rate (mL/min)	Yield^b^ (%)

1	propylene oxide	0.01	0.06	33
2	THF	0.01	0.04	37
3	THF	0.01	0.02	44
4	propylene oxide	0.001	0.06	99
5	THF	0.002	0.06	95
6	cyclohexene	0.002	0.06	–^c^
7	THF	0.002	0.08	68
8	THF	0.003	0.06	50

^a^1.1 equiv of iodine were used. The solvent was dry toluene. ^b^Determined by ^1^H NMR using mesitylene as internal standard. ^c^Mainly *Z*-stilbene was observed.

**Figure 1:**
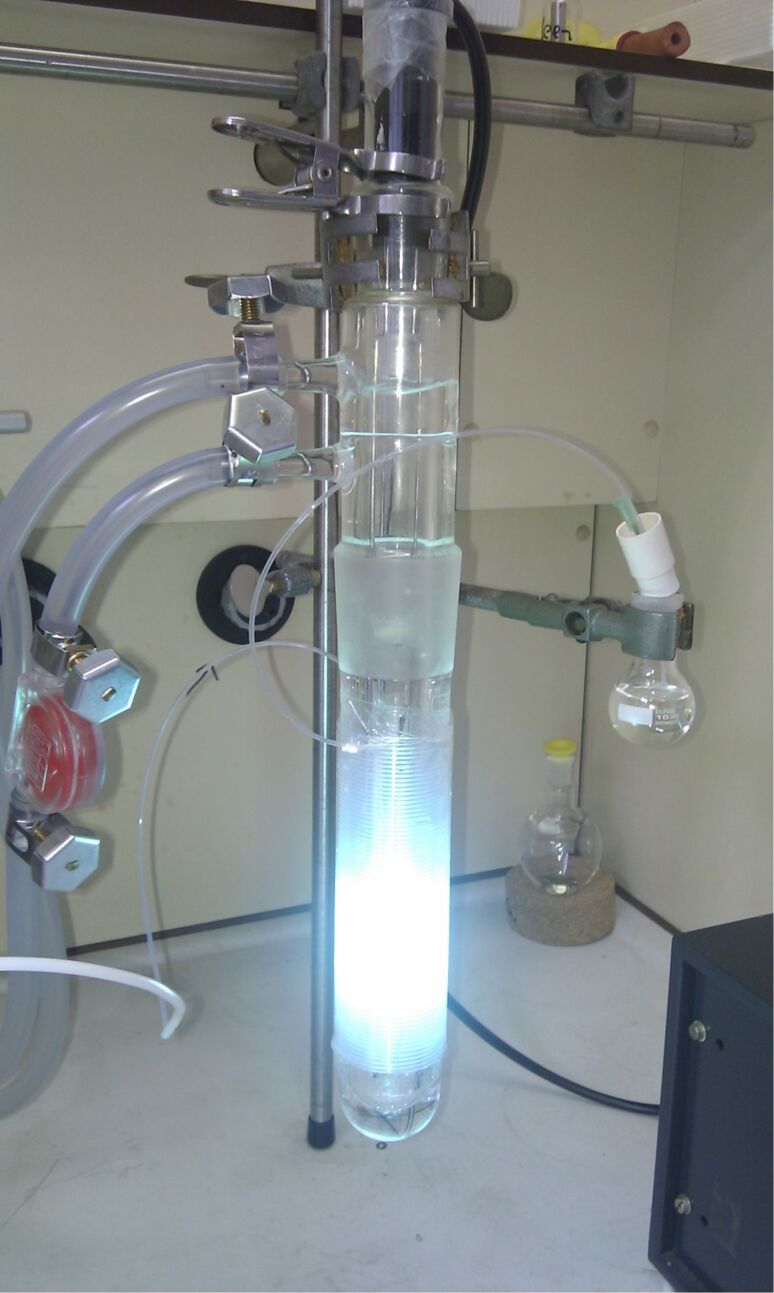
Flow-reactor setup used in the optimization study.

The flow-reactor setup used for the optimization (Table 1) is shown in Figure 1. UV-transparent ethylene propylene copolymer capillary (FEP, outer/inner diameter 1/0.5 mm, total volume 5 mL) was tightly wrapped around the water-cooling unit (Duran glass) of a high-pressure mercury lamp (TQ 150, UV-Consulting Peschl). Further optimization and scope was performed with a similar setup using a bigger capillary (FEP, outer/inner diameter 4/2 mm, total volume 24 mL). For the sake of safety and to enhance the efficiency of irradiation by reflection, the setup was placed into a laboratory Dewar flask and the exposed parts of the setup were covered with aluminium foil (the temperature inside the Dewar was determined to be around 40 °C). The reaction mixture was injected into the system using a syringe pump and collected at the outlet of the tubing into a round bottom flask.

Additionally, we developed a 5-fold bigger setup to enhance the throughput (Table 2). Slightly longer retention times were required, and degassed toluene provided better yields. Finally, phenanthrene (**2a**) was obtained in 94% yield when a flow rate of 0.2 mL/min (retention time of 120 min) was applied (Table 2, entry 4).

**Table 2:** Optimisation of the scale-up setup.

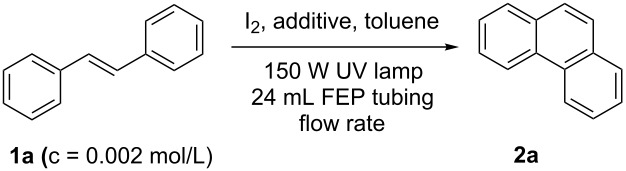			

Entry^a^	Additive (20 equiv)	Flow rate (mL/min)	Yield^b^ (%)

1	propylene oxide	0.2	66
2	THF	0.25	40
3	THF	0.2	85
4^c^	THF	0.2	94

^a^1.1 equiv of iodine were used. Dry toluene was used as solvent. ^b^Determined by ^1^H NMR using mesitylene as internal standard. ^c^Degassed toluene was used.

With the optimised conditions in hand, we explored the scope of the reaction. Although photocyclization of disubstituted olefins in batch was well documented in the literature, only few cases of photocyclization of tri- and tetrasubstituted olefins were reported [16,25]. Therefore, we decided to demonstrate our methodology on both di- and trisubstituted olefins (Table 3). We disclose here the first photocyclization of trisubstituted olefins in flow, giving access to backbone-functionalised phenanthrene derivatives.

**Table 3:** Scope of the photocyclization of stilbene derivatives in continuous flow to give substituted phenanthrenes.

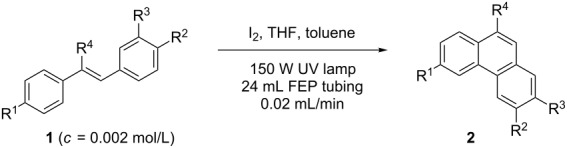			

Entry^a^	Substrate	Product (**2**)	Yield^b^ (%)

1	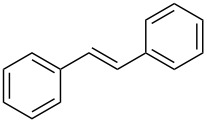	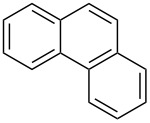 **2a**	64
2	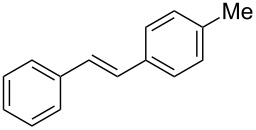	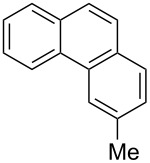 **2b**	64
3	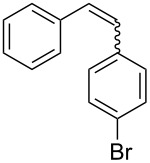	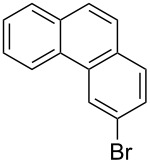 **2c**	61
4	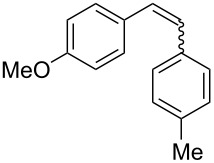	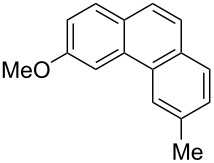 **2d**	77
5	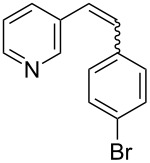	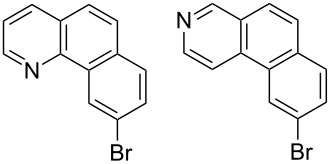 **2e** and **2e’**	31/34
6	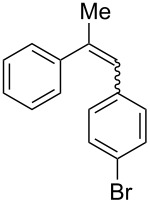	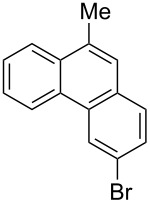 **2f**	77
7	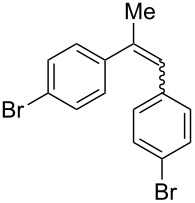	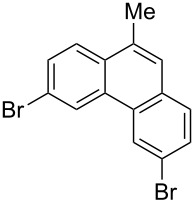 **2g**	89
8	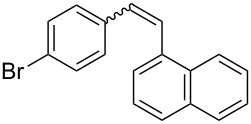	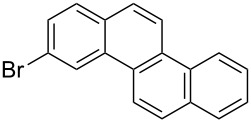 **2h**	64

^a^Reaction conditions: 1.1 equiv iodine, 20 equiv THF, UV-light, 2 h retention time. ^b^Yield after chromatography.

We choose stilbenes with bromide and methyl substituents, as the latter can be used in subsequent oxidation, deprotonation, and radical addition reactions, whereas the former opens access to various functional groups via lithium-halogen exchange or cross-coupling chemistry. Methoxy groups were also tolerated, but nitro- and amino groups containing stilbenes showed low conversion or decomposition. Meta-substituted substrates gave inseparable regioisomers, and ortho-substitution led to low conversion. In the case of substrate **1e**, a separable 1:1 mixture of regioisomers **2e** and **2e’** was obtained (Table 3, entry 5). However, generally a series of stilbenes reacted smoothly to the desired phenantrenes in good yields.

Recently, an unexpected synthesis of [4]helicenes was disclosed [26]. However, no 2-substituted [4]helicenes were synthesised using this method. Therefore, we investigated the photocyclization of the corresponding stilbene derivatives in our flow setup, for both di- and trisubstituted olefins (Table 4).

**Table 4:** Scope: synthesis of [4]helicenes by photocyclization in flow.

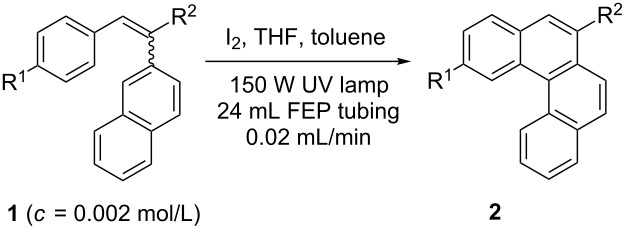				

Entry^a^	Substrate	Product (**2**)		Yield^b^ (%)

1	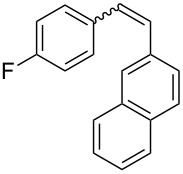		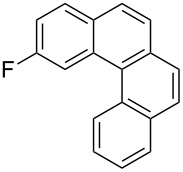 **2i**	85
2	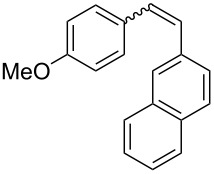		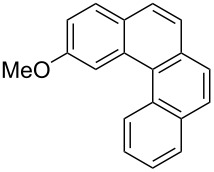 **2j**	75
3	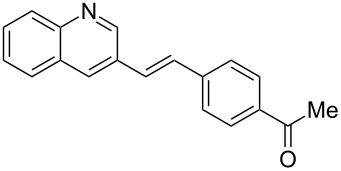		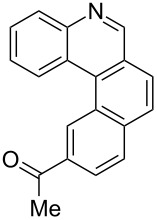 **2k**	74
4^c^	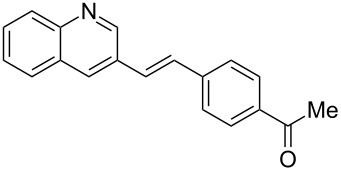		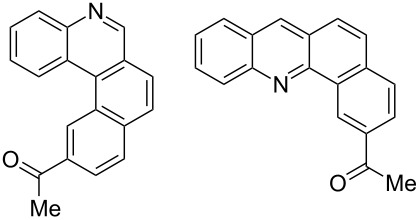 **2k** and **2k’**	40/41
5	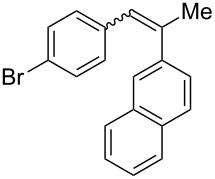		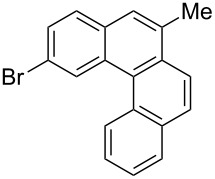 **2l**	75
6	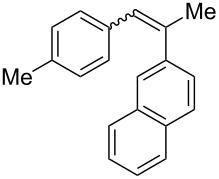		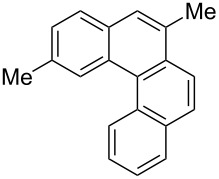 **2m**	73
7	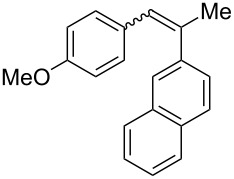		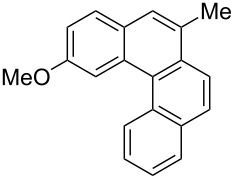 **2n**	99

^a^Reaction conditions: 1.1 equiv iodine, 20 equiv THF, UV-light, 2 h retention time. ^b^Yield after chromatography. ^c^Reaction was performed in dry, degassed acetonitrile.

Again, various functional groups were tolerated in the flow photocyclization. Interestingly, if substrate **1k** was irradiated in toluene a single regioisomer **2k** was isolated (Table 4, entry 3), whereas the reaction in acetonitrile resulted in a separable 1:1 mixture of regioisomers **2k** and **2k’** (Table 4, entry 4).

**Scheme 2:**
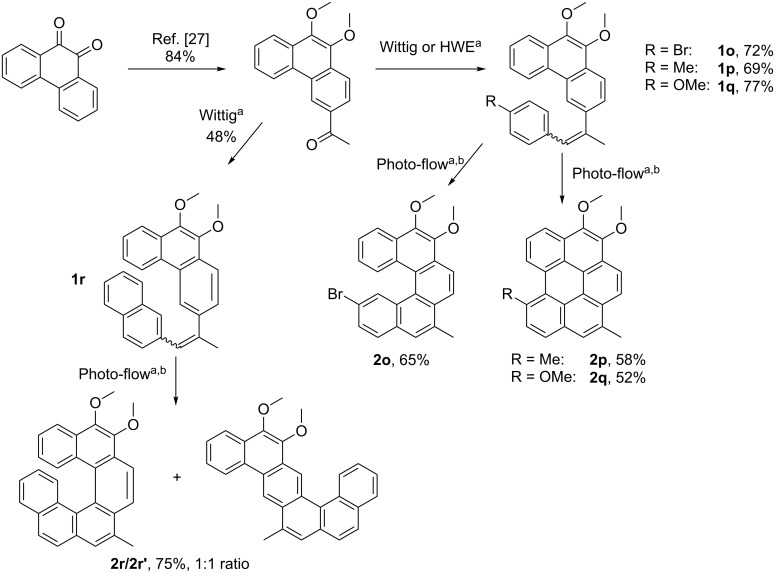
Photo-flow synthesis of [5]- and [6]helicenes. ^a^For experimental details see Supporting Information File 1. ^b^Reaction conditions: 1.1 equiv iodine, 20 equiv THF, UV-light, 2 h retention time.

Finally, we decided to apply our photo-flow methodology in the synthesis of functionalised [5]helicenes and [6]helicenes. We identified 3-acetyl-9,10-dimethoxyphenanthrene [27] as a powerful intermediate for the two-step synthesis of functionalisable helically chiral products. As shown in Scheme 2, Wittig or Horner–Wadsworth–Emmons reactions gave the corresponding olefins **1o–r** in good yields, which were subjected to photocyclization in flow using the optimised conditions for the simpler stilbenes. In the [5]helicene series, in each case only one product was isolated in moderate to good yield. The bromoolefin **1o** gave exclusively the desired helicene **2o**, but in the case of methyl- and methoxyolefins **1p** and **1q**, only the corresponding benzo[*ghi*]perylenes **2p** and **2q** were observed. Benzo[*ghi*]perylenes are typical byproducts observed in the photocyclization of [5]helicene-like molecules. Reactions to obtain selectively helicenes or benzo[*ghi*]perylenes, regardless of the substitution pattern, are still a challenging task. A functionalizable [6]helicene (**2r**) was obtained along with its ribbon-like regioisomer **2r’** in a 1:1 ratio and 75% combined yield.

**Scheme 3:**
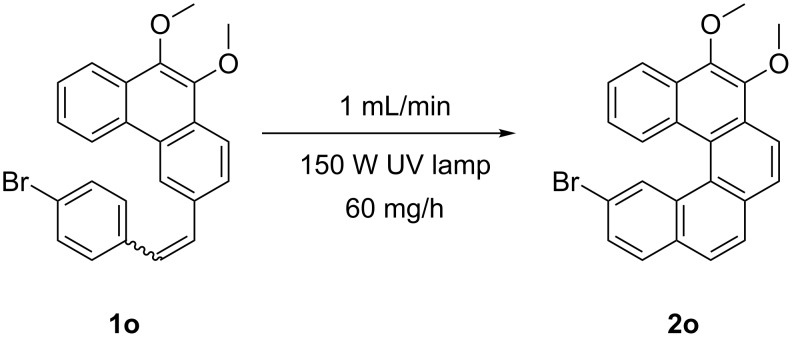
Scale up synthesis of the [5]helicene derivative **2o**.

In order to demonstrate the utility of the flow process we decided to scale up the synthesis of helical compound **2o**. To our delight, we observed that the [5]helicene precursor **1o** underwent photocyclization with considerably shorter retention times compared to the standard stilbene derivatives (Scheme 3). Thus, we prepared the [5]helicene derivative **2o** with up to 60 mg/h (for the full optimisation table, see Supporting Information File 1).

## Conclusion

In summary, we have developed a new photo-flow methodology [28–32] for the synthesis of phenanthrenes and helicenes. Although photocyclization of stilbene derivatives was disclosed more than 40 years ago, this is the first report of UV-light-driven photocyclization in flow. In general phenantrenes as well as [4]-, [5]- and [6]helicenes with different substitution patterns are obtained in good to excellent yields. In addition our first attempts to scale up the flow photocyclization reactions were successful providing the opportunity for multi-gram syntheses.

## Supporting Information

File 1 Experimental part.
