# Supplementary material for: Continuous flow photocyclization of stilbenes – scalable synthesis of functionalized phenanthrenes and helicenes
Source: Beilstein J Org Chem. 2013 Sep 17;9:1883–90. doi: 10.3762/bjoc.9.221 (PMC3778414; doi:10.3762/bjoc.9.221)
Supplement: File 1 — Experimental part. [file Beilstein_J_Org_Chem-09-1883-s001.pdf]

Supporting Information

for

Continuous flow photocyclization of stilbenes – scalable synthesis of functionalized phenanthrenes and helicenes

Quentin Lefebvre, Marc Jentsch and Magnus Rueping*

Address: Institute of Organic Chemistry, RWTH Aachen University, Landoltweg 1, D-52074 Aachen, Germany.

Email: Magnus Rueping* - magnus.rueping@rwth-aachen.de

* Corresponding author

Experimental Part

1. **General information** **S2**

2. **Preparation and characterisation of starting materials** **S3**

3. **Photocyclisation of stilbene derivatives in flow** **S12**

4. **^{1}H and ^{13}C NMR spectra of new compounds** **S22**

1. General information

All reactions were performed with oven-dried glassware and under an inert atmosphere (argon) unless otherwise stated.

THF and toluene were distilled from solvona®/benzoquinone prior to use. Pentane was distilled. Other solvents were used as purchased unless otherwise stated.

Commercial reagents were used as purchased without further purification.

Organic solutions were concentrated under reduced pressure on a Büchi rotary evaporator. Chromatographic purification of products was carried out using Merck Kieselgel 60 silica gel (230–400 mesh). Thin-layer chromatography was carried out using Merck Kieselgel 60 F_{254} (230–400 mesh) fluorescent treated silica and were visualized under UV light (250 and 354 nm) or by staining with aqueous potassium permanganate solution or cerium ammonium molybdate solution.

^{1}H NMR spectra were recorded in deuterated solvents on Bruker or Varian spectrometers at 300, 400 or 600 MHz, with residual protic solvent as the internal standard. ^{13}C NMR spectra were recorded in deuterated solvents on Bruker or Varian spectrometers at 75, 100 or 125 MHz, with the central peak of the deuterated solvent as the internal standard. Chemical shifts (δ) are given in parts per million (ppm), and coupling constants (*J*) are given in Hertz (Hz) rounded to the nearest 0.1 Hz. The ^{1}H NMR spectra are reported as δ/ppm downfield from tetramethylsilane (multiplicity, number of protons, assignment, coupling constant *J*/Hz). The ^{13}C NMR spectra are reported as δ/ppm. Assignments are aided by the use of DEPT-135, COSY, HMQC and HMBC spectra where necessary. IR spectra were recorded on a Perkin Elmer 1760 FT-IR spectrometer, only diagnostic absorbances (λ_{max}) are reported. Low resolution mass spectra were recorded on a Thermo Finnigan SSQ 7000 mass spectrometer (EI). Melting points were recorded on a Büchi Melting Point M-565 apparatus, at ambient pressure and are uncorrected.

2. *Preparation and characterisation of starting materials*

2.1. Preparation of 3-acetyl-9,10-dimethoxyphenanthrene

3-Acetyl-9,10-dimethoxyphenanthrene was synthesised from commercially available phenanthrene-quinone as reported.[1]

2.2. Preparation of Wittig and HWE salts

$ArCH_2PPh_3Br$ salts were prepared following a known procedure.[2]

$ArCH_2P(O)(OMe)_2$ derivatives were prepared in a similar fashion by refluxing the corresponding benzyl bromide derivative in $P(OMe)_3$ overnight.

2.3. Preparation of stilbene derivatives

General procedure A (Wittig reaction)

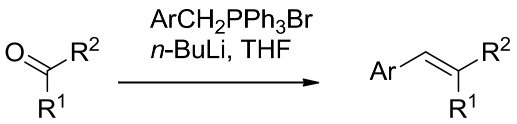

$ArCH_2PPh_3Br$ (2.23 g, 0.55 mmol, 1.1 equiv) was placed in a dry flask under argon. Anhydrous THF (5 mL) was added and the resulting suspension was stirred at 0 °C. n-Butyllithium (0.375 mL, 0.6 mmol, 1.2 equiv) was added and the red mixture was stirred at rt for 2 h. The solution was cooled at 0 °C and the appropriate carbonyl compound (0.5 mmol, 1.0 equiv) was added as a solution in dry THF (1 mL), dropwise over 2 minutes. The mixture was allowed to warm to rt and was stirred overnight. The reaction was quenched by addition of water (10 mL). The biphasic mixture was extracted with ether (20 mL) and the aqueous phase re-extracted with ether (2 × 20 mL). The combined organic layers were washed with brine (40 mL), dried over sodium sulfate, filtered and concentrated in vacuo. The residue was purified by chromatography on silica gel eluting with a pentane/ether gradient to afford the olefin as a mixture of *E* and *Z* isomers. Chemical shifts of the major isomer are given.

[1] D. Z. Wang and T. J. Katz, *J. Org. Chem.*, **2005**, 70, 8497-8502

[2] M. Shi and B. Xu, *J. Org. Chem.*, **2002**, 67, 294-297

General procedure B (HWE reaction)

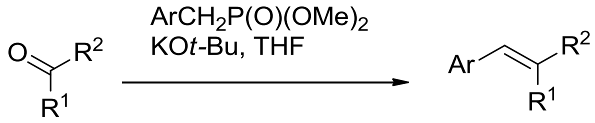

$ArCH_2P(O)(OMe)_2$ (181 mg, 0.65 mmol, 1.3 equiv) and KO*t*-Bu (112 mg, 1.0 mmol, 2.0 equiv) were placed in a dry flask under argon. Anhydrous THF (5 mL) was added at 0 °C and the resulting suspension was stirred at rt for 30 min. The solution was cooled at 0 °C and the appropriate carbonyl compound (0.5 mmol, 1.0 equiv) was added as a solution in dry THF (1 mL), dropwise over 2 minutes. The mixture was allowed to warm to rt and was stirred overnight. The reaction was quenched by addition of water (10 mL). The biphasic mixture was extracted with ether (20 mL) and the aqueous phase re-extracted with ether (2 × 20 mL). The combined organic layers were washed with brine (40 mL), dried over sodium sulfate, filtered and concentrated in vacuo. The residue was purified by chromatography on silica gel eluting with a pentane/ether gradient to afford the olefin as a mixture of *E* and *Z* isomers. Chemical shifts of the major isomer are given.

Preparation and characterisation of *p*-methylstyrylbenzene (1b)

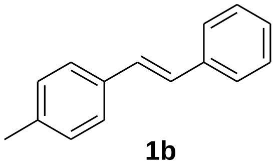

1b

Prepared by a Heck reaction.

Analytical data are in agreement with the literature.[3]

Preparation and characterisation of *p*-bromostyrylbenzene (1c)

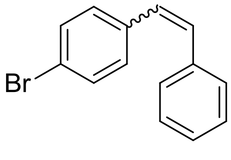

1c

Prepared according to the general procedure **B** on 0.5 mmol scale of benzaldehyde. The crude mixture was purified by column chromatography on silica gel eluting with pentane to afford the title product as a colourless solid (150 mg, 77%). Analytical data are in agreement with the literature.[4]

[3] X. Cui, J. Li, Z.-P. Zhang, Y. Fu, L. Liu and Q.-X. Guo, *J. Org. Chem.*, **2007**, 72, 9342-9345
[4] Y. Leng, F. Yang, K. Wei and Y. Wu, *Tetrahedron*, **2010**, 66, 1244-1248

Preparation and characterisation of 4-(*p*-methoxystyryl)toluene (1d)

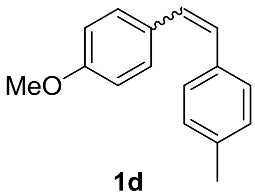

1d

Prepared according to the general procedure **B** on 1 mmol scale of tolualdehyde. The crude mixture was purified by column chromatography on silica gel eluting with pentane/ to afford the title product as a colourless oil (110 mg, 49%). Analytical data are in agreement with the literature.[5]

Preparation and characterisation of 3-(*p*-bromostyryl)pyridine (1e)

Prepared according to the general procedure **A** on 0.5 mmol scale of 3-pyridine carboxaldehyde. The crude mixture was purified by column chromatography on silica gel eluting with pentane/diethyl ether 4:1 to afford the title product as a white solid (93 mg, 72%).

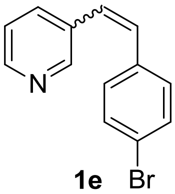

1e

FT-IR ν_{max}(KBr) 3019, 1905, 1753, 1564, 1480, 1413, 1069, 1009, 811, 702 cm^{-1}; **^{1}H NMR** ($CDCl_3$, 600 MHz) δ_H 6.99-7.06 (AA' system, 2H, 2 × C*H*), 7.27 (br. s., 1H, HetAr-C*H*), 7.34 (d, 2H, 2 × Ar-C*H*, *J* 8.5 Hz), 7.46 (d, 2H, 2 × Ar-C*H*, *J* 8.5 Hz), 7.77 (d, 1H, HetAr-C*H*, *J* 8.0 Hz), 8.52 (br. s., 1H, HetAr-C*H*), 8.73 (br. s., 1H, HetAr-C*H*); **^{13}C NMR** ($CDCl_3$, 125 MHz) δ_C 122.0 (*C*quat), 123.8 (*C*quat, broad), 125.7 (2 × *C*H), 128.1 (2 × Ar-*C*H), 129.5 (HetAr-*C*H), 131.2 (2 × Ar-*C*H), 132.7 (HetAr-*C*H), 135.6 (*C*quat.), 148.6 (HetAr-*C*H), 148.8 (HetAr-*C*H); ***m/z*** (EI) 260 ([M]$^{\bullet+}$, 75%), 180([M − Br]$^+$, 100%).

Preparation and characterisation of 1-(*p*-bromophenyl)-2-phenylpropene (1f)

Prepared according to the general procedure **B** on 0.5 mmol of acetophenone. The crude mixture was purified by column chromatography on silica gel eluting with pentane to afford the title product as a colourless solid (125 mg, 92%).

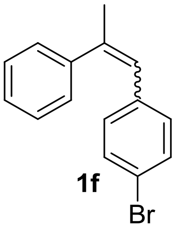

1f

FT-IR ν_{max}(KBr) 1484, 1440, 1072, 1006, 867, 811, 763, 696 cm^{-1}; **^{1}H NMR** ($CDCl_3$, 600 MHz) δ_H 2.45 (d, 3H, C*H*$_3$, *J* 1.5 Hz), 6.65 (s, 1H, C*H*), 7.05 (d, 2H, 2 × Ar-C*H*, *J* 8.5 Hz), 7.41 (dd, 2H, 2 × Ar-C*H*, *J* 1.5 Hz, 6.9 Hz), 7.46 (d, 2H, 2 × Ar-C*H*, *J* 8.5 Hz), 7.51 (m, 1H, Ar-C*H*), 7.55 (m, 2H, 2 × Ar-C*H*); **^{13}C NMR** ($CDCl_3$, 600 MHz) δ_C 27.2 (*C*H$_3$), 120.0 (*C*quat.), 125.5 (*C*H), 127.3 (Ar-*C*H), 128.1 (2 × Ar-

[5] E. Shirakawa, X. Zhang and T. Hayashi, *Angew. Chem. Int. Ed.*, **2011**, 50, 4671-4674

*C*H), 128.7 (2 × Ar-*C*H), 130.6 (2 × Ar-*C*H), 131.1 (2 × Ar-*C*H), 136.0 (*C*quat.), 139.9 (*C*quat.), 141.8 (*C*quat.); ***m/z*** (EI) 274 ($[M]^{•+}$, 60%), 272 ($[M]^{•+}$, 60%) 178 ($[M - CH_3Br]^+$, 100%).

Preparation and characterisation of 1,2-bis(*p*-bromophenyl)propene (1g)

Prepared according to the general procedure **B** on 0.5 mmol of 4'-bromoacetophenone. The crude mixture was purified by column chromatography on silica gel eluting with pentane to afford the title product as a colourless solid (90 mg, 51%).

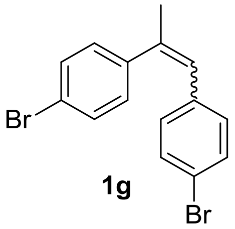

1g

FT-IR ν_{max}(KBr) 2928, 1483, 1395, 1071, 1005, 872, 824 cm^{-1}; **^{1}H NMR** ($CDCl_3$, 600 MHz) δ_H 2.40 (t, 3H, C*H*$_3$, *J* 1.6 Hz), 6.65 (s, 1H, C*H*), 7.04 (dd, 2H, 2 × Ar-C*H*, *J* 1.8, 8.4 Hz), 7.41 (dd, 2H, 2 × Ar-C*H*, *J* 1.8 Hz, 8.4 Hz), 7.46 (dd, 2H, 2 × Ar-C*H*, *J* 1.8, 8.4 Hz), 7.64 (dd, 2H, 2 × Ar-C*H*, *J* 1.8, 8.4 Hz) ; **^{13}C NMR** ($CDCl_3$, 600 MHz) δ_C 26.9 (*C*H$_3$), 120.3 (*C*quat.), 121.2 (*C*quat.), 126.2 (*C*H), 130.0 (2 × Ar-*C*H), 130.6 (2 × Ar-*C*H), 131.2 (2 × Ar-*C*H), 131.9 (2 × Ar-*C*H), 136.2 (*C*quat.), 138.4 (*C*quat.), 140.6 (*C*quat.); ***m/z*** (EI) 354 ($[M]^{•+}$, 50%), 352 ($[M]^{•+}$, 100%), 350 ($[M]^{•+}$, 50%), 192 ($[M - Br_2]^+$, 95%).

Preparation and characterisation of 1-(p-bromostyryl)naphthalene (1h)

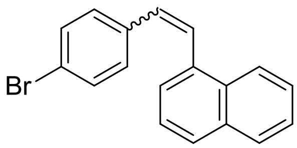

1h

Prepared according to general procedure **B** on 0.1 mmol of 1-naphtaldehyde. The crude mixture was purified by column chromatography on silica gel eluting with pentane to afford the title product as a colourless oil (80 mg, 26%). Analytical data are in agreement with the literature.[6]

Preparation and characterisation of 2-(*p*-fluorostyryl)naphthalene (1i)

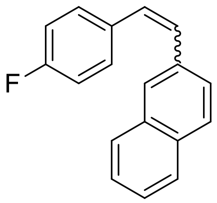

1i

Prepared according to general procedure **B** on 0.5 mmol of 2-naphthaldehyde. The crude mixture was purified by column chromatography on silica gel eluting with pentane to afford the title product as a white solid (73 mg, 59%). Analytical data in agreement with literature.[7]

[6] W. J. Archer, R. Taylor, P. H. Gore and F. S. Kamounah, *J. Chem. Soc., Perkin Trans. 2*, **1980**, 1828-1831
[7] A. R. Ehle, Q. Zhou and M. P. Watson, *Org. Lett.*, **2012**, 14 (5), pp 1202–1205

Preparation and characterisation of 2-(*p*-methoxystyryl)naphthalene 1j

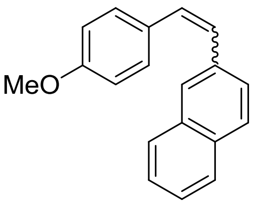

1j

Prepared according to general procedure **A** on 0.5 mmol of 2-naphthaldehyde. The crude mixture was purified by column chromatography on silica gel eluting with pentane/diethyl ether 50:1 to afford the title product as a colourless solid (63 mg, 48%). Analytical data are in agreement with the literature.[7]

Preparation and characterisation of 3-(*p*-acetylstyryl)quinoline (1k)

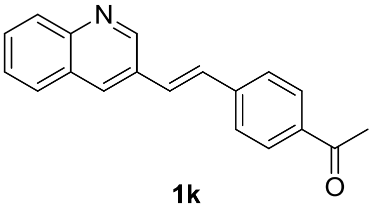

1k

Prepared by a Heck reaction following a reported procedure.[8]

Preparation and characterisation of 1-(*p*-bromophenyl)-2-(2-naphthyl)propene (1l)

Prepared according to general procedure **B** on 0.5 mmol of 2-acetylnaphthalene. The crude mixture was purified by column chromatography on silica gel eluting with pentane to afford the title product as a colourless solid (103 mg, 64%).

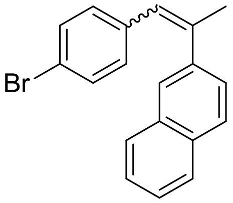

1l

FT-IR ν_{max}(KBr) 3051, 1483, 1005, 872, 806, 742 cm^{-1}; **^{1}H NMR** ($CDCl_3$, 600 MHz) δ_H 2.33 (d, 3H, C*H*$_3$, *J* 1.3 Hz), 6.63 (s, 1H, C*H*), 6.87 (d, 2H, 2 × Ar-C*H*, *J* 8.5 Hz), 7.22 (d, 2H, 2 × Ar-C*H*, *J* 8.5 Hz), 7.28 (dd, 1H, Ar-C*H*, *J* 8.4, 1.6 Hz), 7.50 (dd, 2H, 2 × Ar-C*H*, *J* 6.2 Hz, 3.2 Hz), 7.72 (s, 1H, Ar-C*H*), 7.77 (d, 1H, Ar-C*H*, *J* 8.4 Hz), 7.79-7.85 (m, 2H, 2 × Ar-C*H*) ; **^{13}C NMR** ($CDCl_3$, 600 MHz) δ_C 27.3 (*C*H$_3$), 120.1 (*C*quat.), 125.9 (*C*H), 126.1 (Ar-*C*H), 126.2 (Ar-*C*H), 126.7 (Ar-*C*H), 126.9 (Ar-*C*H), 127.8 (Ar-*C*H), 128.1 (Ar-*C*H), 128.2 (Ar-*C*H), 130.7 (2 × Ar-*C*H), 131.1 (2 × Ar-*C*H), 132.7 (*C*quat.), 133.7 (*C*quat.), 136.6 (*C*quat.), 139.3 (*C*quat.), 139.6 (*C*quat.); ***m/z*** (EI) 324 ([M]$^{\bullet+}$, 100%), 322 ([M]$^{\bullet+}$, 100%) 228 ([M − CH_3Br]$^+$, 90%).

[8] Á. Gordillo, E. de Jesús and C. López-Mardomingo, *Chem. Commun.*, **2007**, 4056-4058

Preparation and characterisation of 1-(*p*-tolyl)-2-(2-naphthyl)propene (1m)

Prepared according to general procedure **A** on 0.5 mmol of 2-acetylnaphthalene. The crude mixture was purified by column chromatography on silica gel eluting with pentane to afford the title product as a colourless oil (55 mg, 43%).

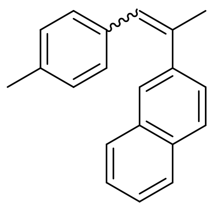

1m

FT-IR ν_{max}(ATR) 2913, 1504, 904, 817, 731 cm^{-1}; **^{1}H NMR** ($CDCl_3$, 400 MHz) δ_H 2.25 (s, 3H, C$\underline{H}_3$), 2.32 (d, 3H, C$\underline{H}_3$, *J* 1.4 Hz), 6.58 (d, 1H, C$\underline{H}$, *J* 1.4 Hz), 6.91 (s, 4H, 4 × Ar-C$\underline{H}$), 7.32 (dd, 1H, Ar-C$\underline{H}$, *J* 1.4, 8.6 Hz), 7.47 (d, 1H, Ar-C$\underline{H}$, *J* 3.3Hz), 7.49 (d, 1H, Ar-C$\underline{H}$, *J* 3.3 Hz), 7.74 (s, 1H, Ar-C$\underline{H}$), 7.75 (d, 1H, Ar-C$\underline{H}$, *J* 6.3 Hz), 7.80 (dd, 1H, Ar-C$\underline{H}$, *J* 3.3, 6.2 Hz), 7.84 (dd, 1H, Ar-C$\underline{H}$, *J* 3.3, 6.2 Hz); **^{13}C NMR** ($CDCl_3$, 100 MHz) δ_C 21.2 ($\underline{C}H_3$), 27.4 ($\underline{C}H_3$), 125.8 (Ar-$\underline{C}$H), 126.0 (Ar-$\underline{C}$H), 126.7 (Ar-$\underline{C}$H), 127.0 (Ar-$\underline{C}$H) 127.3 (Ar-$\underline{C}$H), 127.8 (Ar-$\underline{C}$H), 128.0 (Ar-$\underline{C}$H), 128.1 (Ar-$\underline{C}$H), 128.8 (2 × Ar-$\underline{C}$H), 129.0 (2 × Ar-$\underline{C}$H), 132.6 ($\underline{C}$quat.), 133.8 ($\underline{C}$quat.), 134.7 ($\underline{C}$quat.), 136.0 ($\underline{C}$quat.), 137.6 ($\underline{C}$quat.), 140.0 ($\underline{C}$quat.); ***m/z*** (EI) 258 ($[M]^{\bullet+}$, 100%), 243 ($[M - CH_3]^+$, 60%), 228 ($M - C_2H_6]^+$, 40%).

Preparation and characterisation of 1-(*p*-methoxyphenyl)-2-(2-naphthyl)propene (1n)

Prepared according to general procedure **A** on 0.5 mmol of 2-acetylnaphthaldehyde. The crude mixture was purified by column chromatography on silica gel eluting with pentane/diethyl ether 50:1 to afford the title product as a colourless solid (40 mg, 29%).

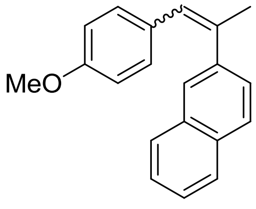

1n

FT-IR ν_{max}(KBr) 2963, 1602, 1505, 1246, 1031, 819 cm^{-1}; **^{1}H NMR** ($CDCl_3$, 600 MHz) δ_H 2.29 (d, 3H, C$\underline{H}_3$, *J* 1.5 Hz), 3.71 (s, 3H, OC$\underline{H}_3$), 6.52 (s, 1H, C$\underline{H}$), 6.62 (d, 2H, 2 × Ar-C$\underline{H}$, *J* 8.9 Hz), 6.92 (d, 2H, 2 × Ar-C$\underline{H}$, *J* 8.9 Hz), 7.30 (dd, 1H, Ar-C$\underline{H}$, *J* 8.4, 1.7 Hz), 7.46 (dd, 2H, 2 × Ar-C$\underline{H}$, *J* 6.2, 3.3), 7.72 (s, 1H, Ar-C$\underline{H}$), 7.74 (d, 1H, Ar-C$\underline{H}$, *J* 8.4 Hz), 7.78 (dd, 1H, Ar-C$\underline{H}$, *J* 6.2, 3.3 Hz), 7.81 (dd, 1H, Ar-C$\underline{H}$, *J* 6.2, 3.3 Hz); **^{13}C NMR** ($CDCl_3$, 150 MHz) δ_C 27.3 ($\underline{C}H_3$), 55.2 (O$\underline{C}H_3$), 113.5 (2 × Ar-$\underline{C}$H), 125.8 ($\underline{C}$H), 126.0 (Ar-$\underline{C}$H), 126.5 (Ar-$\underline{C}$H), 126.7 (Ar-$\underline{C}$H), 127.3 (Ar-$\underline{C}$H), 127.9 (Ar-$\underline{C}$H), 128.0 (Ar-$\underline{C}$H), 128.1 (Ar-$\underline{C}$H), 130.3 (2 × Ar-$\underline{C}$H), 132.6 ($\underline{C}$quat.), 133.8 ($\underline{C}$quat.), 136.6 ($\underline{C}$quat.), 140.1 ($\underline{C}$quat.), 158.1 ($\underline{C}$quat.); ***m/z*** (EI) 274 ($[M]^{\bullet+}$, 100%).

Preparation and characterisation of 1-(*p*-bromophenyl)-2-[3-(9,10-dimethoxyphenanthrene)]propene (1o)

p-$BrPhCH_2PPh_3Br$ (563 mg, 1.1 mmol, 1.1 equiv), 18-crown-6 (11 mg, 0.04 mmol, 0.04 equiv) and KOtBu (135 mg, 1.2 mmol, 1.2 equiv) were placed in a dry flask under argon. Anhydrous toluene (10 mL) was added at 0 °C and the resulting suspension was stirred at rt for 2 h. The solution was cooled at 0 °C and 3-acetyl-9,10-dimethoxyphenanthrene (280 mg, 1.0 mmol, 1.0 equiv) was added. The mixture was allowed to warm to rt and was then refluxed overnight. The reaction mixture was allowed to cool at rt and was quenched by addition of water (10 mL). The biphasic mixture was extracted with ether (20 mL) and the aqueous phase re-extracted with ether (2 × 20 mL). The combined organic layers were washed with brine (40 mL), dried over sodium sulfate, filtered and concentrated in vacuo. The residue was purified by chromatography on silica gel eluting with a pentane/ether gradient to afford the title product as a yellow oil (113 mg, 26%).

E-enriched substrate could be obtained as a white foam in 72% yield using general procedure **B** on 0.5 mmol of 3-acetyl-9,10-dimethoxyphenanthrene.

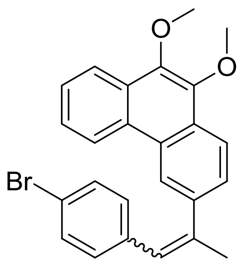

1o

FT-IR ν_{max}(ATR) 2938, 1607, 1488, 1452, 1326, 1072, 832, 767, 732 cm^{-1}; **^{1}H NMR** ($CDCl_3$, 400 MHz) δ_H 2.47 (s, 1H, $C\underline{H}_3$), 4.16 (s, 6H, 2 × $OC\underline{H}_3$), 6.99 (s, 1H, C*H̲*), 7.33 (d, 2H, 2 × Ar-C*H̲*, *J* 8.4 Hz), 7.56 (d, 2H, 2 × Ar-C*H̲*, *J* 8.4 Hz), 7.65-7.71 (m, 2H, Ar-$C\underline{H}_{b'}$ and Ar-$C\underline{H}_{c'}$), 7.85 (dd, 1H, Ar-$C\underline{H}_c$, *J* 8.5, 1.5 Hz), 8.29 (d, 1H, Ar-$C\underline{H}_d$, *J* 8.5 Hz), 8.32 (dd, 1H, Ar-$C\underline{H}_{d'}$, *J* 7.5, 1.7 Hz), 8.75 (d, 1H, Ar-$C\underline{H}_{a'}$, *J* 7.5 Hz), 8.79 (d, 1H, Ar-$C\underline{H}_a$, *J* 1.5 Hz);

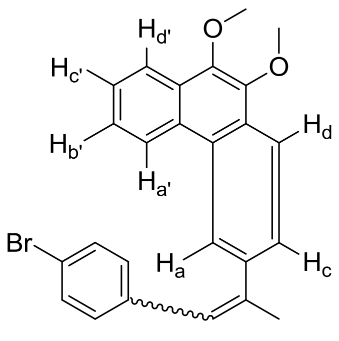

^{13}C NMR ($CDCl_3$, 100 MHz) δ_C 17.9 ($\underline{C}H_3$), 61.1 (2 × $O\underline{C}H_3$), 120.1 (C̲H), 120.5 (C̲quat.), 122.3 (Ar-C̲H), 122.4 (Ar-C̲H), 122.7 (Ar-C̲H), 125.1 (Ar-C̲H), 126.0 (Ar-C̲H), 127.1 (Ar-C̲H), 127.2 (Ar-C̲H), 128.5 (C̲quat.), 128.7 (C̲quat.), 128.8 (C̲quat.), 129.5 (C̲quat.), 131.0 (2 × Ar-C̲H), 131.5 (2 × Ar-C̲H), 137.3 (C̲quat.), 138.6 (C̲quat.), 141.2 (C̲quat.), 144.0 (C̲quat.), 144.1 (C̲quat.); ***m/z*** (EI) 434 ($[M]^{\bullet+}$, 100%), 432 ($[M]^{\bullet+}$, 100%).

Preparation and characterisation of 1-(*p*-tolyl)-2-[3-(9,10-dimethoxyphenanthrene)]propene (1p)

Prepared according to general procedure **A** on 0.5 mmol of 3-acetyl-9,10-dimethoxyphenanthrene. The crude mixture was purified by column chromatography on silica gel eluting with pentane/diethyl ether 20:1 to afford the title product as a colourless oil (127 mg, 69%).

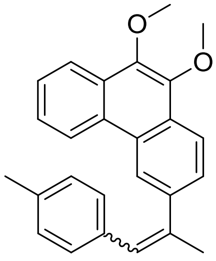

1p

FT-IR ν_{max}(ATR) 2940, 1607, 1445, 1327, 1071, 831, 764, 732 cm^{-1}; **^{1}H NMR** ($CDCl_3$, 400 MHz) δ_H 2.46 (s, 3H, C*H*$_3$), 2.53 (d, 3H, C*H*$_3$, *J* 1.3 Hz), 4.17 (s, 6H, 2 × OC*H*$_3$), 7.09 (s, 1H, C*H*), 7.28 (d, 2H, 2 × Ar-C*H*, *J* 7.9 Hz), 7.41 (d, 2H, 2 × Ar-C*H*, *J* 7.9 Hz), 7.67-7.70 (m, 2H, C*H*$_{b'}$ and C*H*$_{c'}$), 7.89 (dd, 1H, C*H*$_c$, *J* 8.6, 1.8 Hz), 8.30 (d, 1H, C*H*$_d$, *J* 8.6 Hz), 8.31-8.34 (m, 1H, C*H*$_{d'}$), 8.76-8.79 (m, 1H, C*H*$_{a'}$), 8.82 (d, 1H, C*H*$_a$, *J* 1.8 Hz); **^{13}C NMR** ($CDCl_3$, 100 MHz) δ_C 17.9 (*C*H$_3$), 21.4 (*C*H$_3$), 61.1 (2 × O*C*H$_3$), 120.0 (*C*H), 122.2 (Ar-*C*H), 122.3 (Ar-*C*H), 122.7 (Ar-*C*H), 125.2 (Ar-*C*H), 125.9 (Ar-*C*H), 127.0 (Ar-*C*H), 128.0 (*C*quat.), 128.4 (Ar-*C*H), 128.7 (*C*quat.), 128.9 (*C*quat.), 129.1 (2 × Ar-*C*H), 129.3 (2 × Ar-*C*H), 129.5 (*C*quat.), 135.6 (*C*quat.), 136.4 (*C*quat.), 137.0 (*C*quat.), 141.7 (*C*quat.), 144.0 (*C*quat.), 144.1 (*C*quat.); ***m/z*** (EI) 368 ([M]$^{\bullet+}$, 100%).

Preparation and characterisation of 1-(*p*-methoxyphenyl)-2-[3-(9,10-dimethoxyphenanthrene)]-propene (1q)

Prepared according to general procedure **A** on 0.5 mmol of 3-acetyl-9,10-dimethoxyphenanthrene. The crude mixture was purified by column chromatography on silica gel eluting with pentane/diethyl ether 10:1 to afford the title product as a colourless oil (148 mg, 77%).

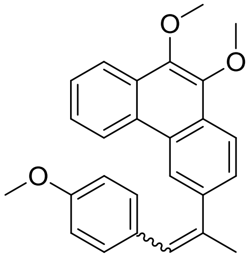

1q

FT-IR ν_{max}(ATR) 2941, 1606, 1507, 1247, 1072, 831, 768 cm^{-1}; **^{1}H NMR** ($CDCl_3$, 400 MHz) δ_H 2.53 (d, 3H, C*H*$_3$, *J* 1.4 Hz), 3.89 (s, 3H, OC*H*$_3$), 4.18 (s, 6H, 2 × OC*H*$_3$), 7.01 (d, 2H, 2 × Ar-C*H*, *J* 8.7 Hz), 7.07 (s, 1H, C*H*), 7.45 (d, 2H, 2 × Ar-C*H*, *J* 8.7 Hz), 7.66-7.72 (m, 2H, C*H*$_{b'}$ and C*H*$_{c'}$), 7.90 (dd, 1H, C*H*$_c$, *J* 8.5, 1.8 Hz), 8.31 (d, 1H, C*H*$_d$, *J* 8.5 Hz), 8.34 (dd, 1H, C*H*$_{d'}$, *J* 7.5, 2.0 Hz), 8.79 (m, 1H, C*H*$_{a'}$, J 8.3, 1.0 Hz), 8.83 (d, 1H, C*H*$_a$, *J* 1.8 Hz); **^{13}C NMR** ($CDCl_3$, 100 MHz) δ_C 17.9 (*C*H$_3$), 55.3 (O*C*H$_3$), 61.1 (2 × O*C*H$_3$), 113.8 (2 × Ar-*C*H), 119.9 (*C*H), 122.2 (Ar-*C*H), 122.3 (Ar-*C*H), 122.7 (Ar-*C*H), 125.2 (Ar-*C*H), 125.8 (Ar-*C*H), 127.0 (Ar-*C*H), 128.0 (Ar-*C*H), 128.1 (*C*quat.), 128.7 (*C*quat.), 128.8 (*C*quat.), 129.5 (*C*quat.), 130.5 (2 × Ar-*C*H), 131.0

($\underline{C}$quat.), 136.1 ($\underline{C}$quat.), 141.7 ($\underline{C}$quat.), 143.9 ($\underline{C}$quat.), 144.0 ($\underline{C}$quat.), 158.3 ($\underline{C}$quat.); ***m/z*** (EI) 384 ($[M]^{\bullet+}$, 100%).

Preparation and characterisation of 1-(2-naphtyl)-2-[3-(9,10-dimethoxyphenanthrene)]propene (1r)

Prepared according to general procedure **A** on 0.5 mmol of 3-acetyl-9,10-dimethoxyphenanthrene. The crude mixture was purified by column chromatography on silica gel eluting with pentane/diethyl ether 20:1 to afford the title product as a colourless oil (97 mg, 48%).

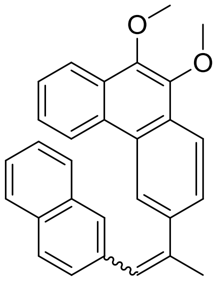

1r

FT-IR ν_{max}(ATR) 2910, 2109, 1508, 965, 819 cm^{-1}; **^{1}H NMR** ($CDCl_3$, 400 MHz) δ_H 2.62 (d, 3H, C$\underline{H}_3$, *J* 1.3 Hz), 4.20 (s, 3H, OC$\underline{H}_3$), 4.21 (s, 3H, OC$\underline{H}_3$), 7.28 (d, 1H, C$\underline{H}$, *J* 1.3 Hz), 7.55-7.58 (m, 2H, 2 × Ar-C$\underline{H}$), 7.65-7.69 (m, 2H, 2 × Ar-C$\underline{H}$), 7.70-7.73 (m, 2H, 2 × Ar-C$\underline{H}$), 7.93-7.97 (m, 4H, 4 × Ar-C$\underline{H}$), 8.35 (d, 1H, Ar-C$\underline{H}$, *J* 8.5 Hz), 8.36 (dd, 1H, Ar-C$\underline{H}$, *J* 7.5, 1.9 Hz), 8.80-8.83 (m, 1H, Ar-C$\underline{H}$), 8.89 (d, 1H, C$\underline{H}$, *J* 1.6 Hz); **^{13}C NMR** ($CDCl_3$, 100 MHz) δ_C 18.0 ($\underline{C}H_3$), 61.1 (2 × O$\underline{C}H_3$), 120.1 ($\underline{C}$H), 122.3 (Ar-$\underline{C}$H), 122.4 (Ar-$\underline{C}$H), 122.8 (Ar-$\underline{C}$H), 125.2 (Ar-$\underline{C}$H), 125.9 (Ar-$\underline{C}$H), 126.3 (Ar-$\underline{C}$H), 127.0 (Ar-$\underline{C}$H), 127.7 (Ar-$\underline{C}$H), 127.8 (2 × Ar-$\underline{C}$H), 127.8 (Ar-$\underline{C}$H), 128.0 (Ar-$\underline{C}$H), 128.1 (Ar-$\underline{C}$H), 128.4 ($\underline{C}$quat.), 128.5 (Ar-$\underline{C}$H) 128.7 ($\underline{C}$quat.), 128.9 ($\underline{C}$quat.), 129.5 ($\underline{C}$quat.), 132.3 ($\underline{C}$quat.), 132.5 ($\underline{C}$quat.), 136.0 ($\underline{C}$quat.), 138.2 ($\underline{C}$quat.), 141.5 ($\underline{C}$quat.), 144.0 ($\underline{C}$quat.), 144.0 ($\underline{C}$quat.); ***m/z*** (EI) 404 ($[M]^{\bullet+}$, 100%), 389 ($[M – CH_3]^{+}$, 40%).

3. Photocyclisation of stilbene derivatives in flow

General procedure for photo-flow experiments

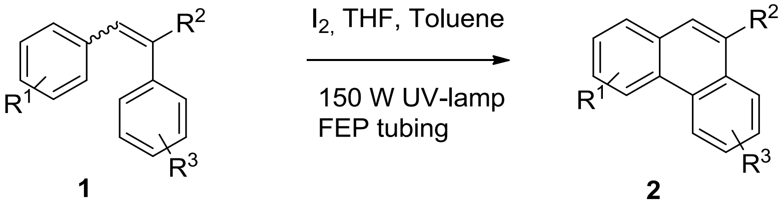

The stilbene derivative **1** (1.0 equiv, $c = 0.002$ mol·L^{-1}) and iodine (1.1 equiv) were dissolved in dry and degassed toluene, and dry THF was added (20 equiv). The red solution was transferred into a 24 mL or 60 mL syringe and pumped into the photo-flow setup via a syringe pump (the flow rate was adapted to the setup to maintain a two-hour retention time). After all the solution was pumped into the system, dry and degassed toluene was pumped with the same flow rate for two hours to flush the tubing. The collected solution was quenched by aqueous sodium thiosulfate (30 mL). The aqueous phase was extracted with toluene (30 mL), and the combined organic layers were washed with brine (50 mL), dried over sodium sulfate, filtered and concentrated in vacuo. The residue was purified by chromatography on silica gel eluting with a pentane/diethyl ether mixture to afford the phenanthrene derivative **2**.

Preparation and characterisation of phenanthrene (2a)

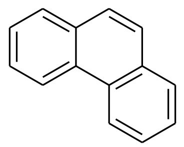

2a

Prepared according to the general procedure on 0.053 mmol scale of stilbene. The crude mixture was purified by column chromatography on silica gel eluting with pentane to afford the title product as a colourless solid (6 mg, 64%). Analytical data are in agreement with the literature.[2]

Preparation and characterisation of 3-methylphenanthrene (2b)

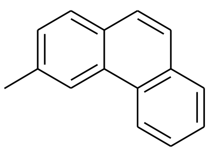

2b

Prepared according to the general procedure on 0.049 mmol scale of *p*-methylstyrylbenzene. The crude mixture was purified by column chromatography on silica gel eluting with pentane to afford the title product as a colourless solid (6 mg, 64%). Analytical data are in agreement with the literature.[9]

[9] S. Paul, R. Jana and J. K. Ray, *Synlett*, **2010**, 10, 1463-1468

Preparation and characterisation of 3-bromophenanthrene (2c)

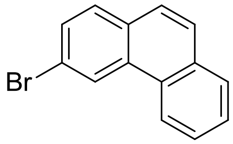

2c

Prepared according to the general procedure on 0.032 mmol scale of *p*-bromostyrylbenzene. The crude mixture was purified by column chromatography on silica gel eluting with pentane to afford the title product as a colourless solid (6 mg, 61%). Analytical data are in agreement with the literature.[10]

Preparation and characterisation of 3-methoxy-6-methylphenanthrene (2d)

Prepared according to the general procedure on 0.048 mmol scale of 4-(*p*-methoxystyryl)toluene. The crude mixture was purified by column chromatography on silica gel eluting with pentane to afford the title product as a colourless solid (8.2 mg, 77%).

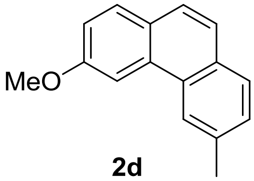

2d

m.p. 98-100 ºC; **FT-IR** ν_{max}(KBr) 2925, 1608, 1448, 1222, 1027, 836 cm^{-1}; **^{1}H NMR** ($CDCl_3$, 600 MHz) δ_H 2.63 (s, 3H, C<u>*H*</u>$_3$), 4.04 (s, 3H, OC<u>*H*</u>$_3$), 7.23 (dd, 1H, Ar-C<u>*H*</u>, *J* 8.7, 2.5 Hz), 7.42 (dd, 1H, Ar-C<u>*H*</u>, *J* 8.2, 1.5 Hz), 7.60 (AA' system, 2H, 2 × Ar-C<u>*H*</u>), 7.77 (d, 1H, Ar-C<u>*H*</u>, *J* 8.2 Hz), 7.79 (d, 1H, Ar-C<u>*H*</u>, *J* 8.7 Hz), 8.04 (d, 1H, Ar-C<u>*H*</u>, *J* 2.5 Hz), 8.38 (s, 1H, Ar-C<u>*H*</u>); **^{13}C NMR** ($CDCl_3$, 150 MHz) δ_C 25.8 (<u>*C*</u>H$_3$), 55.7 (O<u>*C*</u>H$_3$), 104.0 (Ar-<u>*C*</u>H), 116.8 (Ar-<u>*C*</u>H), 122.5 (Ar-<u>*C*</u>H), 124.5 (Ar-<u>*C*</u>H), 125.7 (Ar-<u>*C*</u>H), 127.0 (<u>*C*</u>quat.), 128.5 (Ar-<u>*C*</u>H), 128.6 (Ar-<u>*C*</u>H), 129.9 (<u>*C*</u>quat.), 130.1 (Ar-<u>*C*</u>H), 130.5 (<u>*C*</u>quat.), 131.4 (<u>*C*</u>quat.), 135.9 (<u>*C*</u>quat.), 158.4 (<u>*C*</u>quat.); ***m/z*** (EI) 222 ([M]$^{\bullet+}$, 100%), 109 ([M − CH_3]$^+$, 20%).

Preparation and characterisation of 6-bromo-4-azaphenanthrene (2e) and 6-bromo-2-azaphenanthrene (2e')

Prepared according to the general procedure on 0.034 mmol scale of 3-(*p*-bromostyryl)pyridine. The crude mixture was purified by column chromatography on silica gel eluting with pentane/diethyl ether 4:1. The first isomer was an orange solid (2.7 mg, 31%). The second isomer was an orange solid (3.0 mg, 34%).

[10] A. N. Nesmeyanov, T. P. Tolstaya, L. N. Vanchikova and A. V. Petrakov, *Bull. Acad. Sci. USSR*, **1980**, 29, 1789-1792

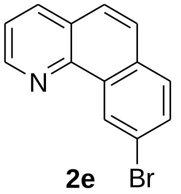

2e

m.p. 89-92 °C; **FT-IR** ν_{max}(KBr) 2921, 1584, 1440, 837, 709 cm^{-1}; **^{1}H NMR** ($CDCl_3$, 600 MHz) δ_H 7.56 (dd, 1H, Ar-C*H*, *J* 8.0, 4.3 Hz), 7.72 (d, 1H, Ar-C*H*, *J* 8.8 Hz), 7.78 (d, 1H, Ar-C*H*, *J* 8.8 Hz), 7.79 (s, 2H, 2 × Ar-C*H*), 8.20 (dd, 1H, Ar-C*H*, *J* 8.0, 1.7 Hz), 9.01 (dd, 1H, Ar-C*H*, *J* 4.3, 1.7 Hz), 9.47 (s, 1H, Ar-C*H*); **^{13}C NMR** ($CDCl_3$, 150 MHz) δ_C 121.7 (*C*quat.), 122.5 (Ar-*C*H), 126.0 (Ar-*C*H), 126.8 (*C*quat.), 127.3 (Ar-*C*H), 127.3 (Ar-*C*H), 129.5 (Ar-*C*H), 131.2 (Ar-*C*H), 132.2 (Ar-*C*H), 132.2 (*C*quat.), 133.0 (*C*quat.), 136.0 (Ar-*C*H), 145.6 (*C*quat.), 149.3 (Ar-*C*H); ***m/z*** (EI) 259 ($[M]^{\bullet+}$, 100%), 257 ($[M]^{\bullet+}$, 100%).

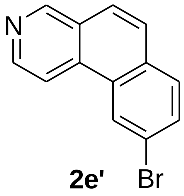

2e'

m.p. 140-143 °C; **FT-IR** ν_{max}(KBr) 2924 , 1584, 1023, 840 cm^{-1}; **^{1}H NMR** ($CDCl_3$, 600 MHz) δ_H 7.82 (AA' system, 2H, 2 × Ar-C*H*), 7.83 (AA' system, 2H, 2 × Ar-C*H*), 8.35 (d, 1H, Ar-C*H*, *J* 5.7 Hz), 8.79 (d, 1H, Ar-C*H*, *J* 5.7 Hz), 8.81 (s, 1H, Ar-C*H*), 9.27 (s, 1H, Ar-C*H*); **^{13}C NMR** ($CDCl_3$, 150 MHz) δ_C 116.1 (*C*quat.), 121.7 (*C*quat.), 125.4 (Ar-*C*H), 126.3 (Ar-*C*H), 128.0 (Ar-*C*H), 130.1 (*C*quat.), 130.4 (Ar-*C*H), 132.1 (2 × Ar-*C*H), 132.2 (*C*quat.), 133.9 (*C*quat.), 145.6 (Ar-*C*H), 152.0 (Ar-*C*H); ***m/z*** (EI) 259 ($[M]^{\bullet+}$, 100%), 257 ($[M]^{\bullet+}$, 100%).

Preparation and characterisation of 3-bromo-9-methylphenanthrene (2f)

Prepared according to the general procedure on 0.048 mmol of 1-(*p*-bromophenyl)-2-phenylpropene. The crude mixture was purified by column chromatography on silica gel eluting with pentane to afford the title product as a colourless solid that melts at room temperature (10 mg, 77%).

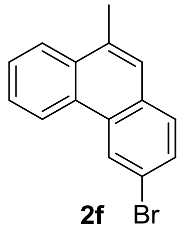

2f

FT-IR ν_{max}(ATR) 2896, 1589, 866, 746 cm^{-1}; **^{1}H NMR** ($CDCl_3$, 400 MHz) δ_H 2.72 (d, 3H, C*H*$_3$, *J* 1.1 Hz), 7.53 (s, 1H, C*H*), 7.63-7.70 (m, 4H, 4 × Ar-C*H*), 8.04-8.08 (m, 1H, Ar-C*H*), 8.61-8.65 (m, 1H, Ar-C*H*), 8.77 (dd, 1H, Ar-C*H*, *J* 1.6, 0.6 Hz); **^{13}C NMR** ($CDCl_3$, 100 MHz) δ_C 20.2 (*C*H$_3$), 120.1 (*C*quat), 123.2 (Ar-*C*H), 124.9 (Ar-*C*H), 125.5 (Ar-*C*H), 126.2 (Ar-*C*H), 126.7 (Ar-*C*H), 127.3 (Ar-*C*H), 129.5 (Ar-*C*H), 129.8 (Ar-*C*H), 130.7 (*C*quat.), 131.1 (*C*quat.), 131.3 (*C*quat.), 132.4 (*C*quat.), 133.3 (*C*quat.); ***m/z*** (EI) 272 ($[M]^{\bullet+}$, 100%), 270 ($[M]^{\bullet+}$, 100%), 191 ($[M - Br]^{+}$, 55%).

Preparation and characterisation of 3,6-dibromo-9-methylphenanthrene (2g)

Prepared according to the general procedure on 0.048 mmol of 1,2-bis(*p*-bromophenyl)propene. The crude mixture was purified by column chromatography on silica gel eluting with pentane to afford the title product as a colourless solid (15 mg, 89%).

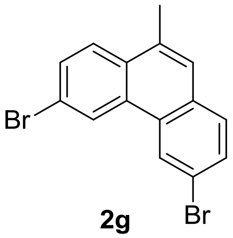

2g

m.p. 142-144 ºC; **FT-IR** ν_{max}(KBr) 2922, 1584, 1488, 1015, 873, 802 cm^{-1}; **^{1}H NMR** ($CDCl_3$, 400 MHz) δ_H 2.68 (d, 3H, C$\underline{H}_3$, *J* 1.1 Hz), 7.51 (s, 1H, C$\underline{H}$), 7.66-7.67 (m, 2H, 2 × Ar-C$\underline{H}$), 7.73 (dd, 1H, Ar-C$\underline{H}$, *J* 8.8, 2.0 Hz), 7.89 (d, 1H, Ar-C$\underline{H}$, J 8.8 Hz), 8.64 (s, 1H, Ar-C$\underline{H}$), 8.70 (d, 1H, Ar-C$\underline{H}$, *J* 2.0 Hz); **^{13}C NMR** ($CDCl_3$, 100 MHz) δ_C 20.2 ($\underline{C}H_3$), 120.1 ($\underline{C}$quat), 123.2 (Ar-$\underline{C}$H), 124.9 (Ar-$\underline{C}$H), 125.5 (Ar-$\underline{C}$H), 126.2 (Ar-$\underline{C}$H), 126.7 (Ar-$\underline{C}$H), 127.3 (Ar-$\underline{C}$H), 129.5 (Ar-$\underline{C}$H), 129.8 (Ar-$\underline{C}$H), 130.7 ($\underline{C}$quat.), 131.1 ($\underline{C}$quat.), 131.3 ($\underline{C}$quat.), 132.4 (2 × $\underline{C}$quat.), 133.3 (2 × $\underline{C}$quat.);; ***m/z*** (EI) 352 ($[M]^{\bullet+}$, 50%), 350 ($[M]^{\bullet+}$, 100%), 348 ($[M]^{\bullet+}$, 50%), 190 ($[M - Br_2]^+$, 25%).

Preparation and characterisation of 3-bromochrysene (2h)

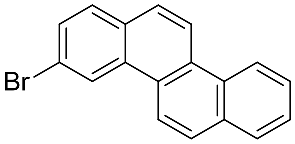

2h

Prepared according to the general procedure on 0.048 mmol of 1-(p-bromostyryl)naphthalene. The crude mixture was purified by column chromatography on silica gel eluting with pentane to afford the title product as a colourless solid (9.5 mg, 64%). Analytical data are in agreement with the literature.[6]

Preparation and characterisation of 2-fluoro[4]helicene (2i)

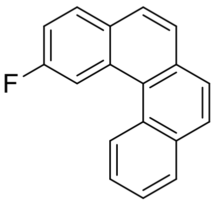

2i

Prepared according to the general procedure on 0.048 mmol of 2-(*p*-flurostyryl)naphthalene. The crude mixture was purified by column chromatography on silica gel eluting with pentane to afford the title product as a colourless solid (10 mg, 85%). Analytical data are in agreement with the literature.[11]

[11] A. V. Bedekar, A. R. Chaudhary, M. S. Sundar and M. Rajappa, *Tetrahedron Lett.*, **2013**, 54, 392–396

Preparation and characterisation of 2-methoxy[4]helicene (2j)

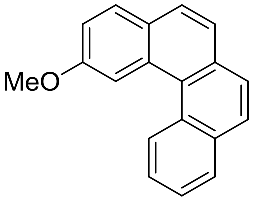

2j

Prepared according to the general procedure on 0.044 mmol of 2-(*p*-methoxystyryl)naphthalene. The crude mixture was purified by column chromatography on silica gel eluting with pentane to afford the title product as a colourless solid (8.5 mg, 75%). Analytical data are in agreement with the literature.[9]

Preparation and characterisation of 2-acetyl[4](8-azahelicene) (2k)

Prepared according to the general procedure on 0.04 mmol of 3-(*p*-acetylstyryl)quinoline. The crude mixture was purified by column chromatography on silica gel eluting with pentane/ethyl acetate 1:1 to 1:4 to afford the title product as a colourless solid (8 mg, 74%).

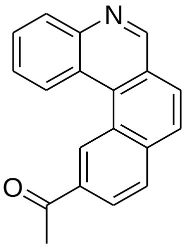

2k

m.p. 120-124 °C; **FT-IR** ν_{max}(KBr) 2924, 1668, 1256, 772 cm^{-1}; **^{1}H NMR** ($CDCl_3$, 600 MHz) δ_H 2.83 (s, 1H, $C\underline{H}_3$), 7.82-7.89 (m, 2H, 2 × Ar-C$\underline{H}$), 8.07 (AA' system, 2H, 2 × Ar-C$\underline{H}$), 8.15 (d, 1H, Ar-C$\underline{H}$, *J* 8.4 Hz), 8.29 (dd, 1H, Ar-C$\underline{H}$, *J* 8.4, 1.6 Hz), 8.37 (dd, 1H, Ar-C$\underline{H}$, *J* 8.1, 1.4 Hz), 9.06 (d, 1H, Ar-C$\underline{H}$, *J* 8.1 Hz), 9.39 (s, 1H, Ar-C$\underline{H}$), 9.83 (s, 1H, Ar-C$\underline{H}$); **^{13}C NMR** ($CDCl_3$, 150 MHz) δ_C 27.1 ($\underline{C}H_3$), 124.4 ($\underline{C}$quat.), 125.6 ($\underline{C}$quat.), 126.5 (Ar-$\underline{C}$H), 126.8 (Ar-$\underline{C}$H), 127.7 (Ar-$\underline{C}$H), 127.9 (Ar-$\underline{C}$H), 128.4 (Ar-$\underline{C}$H), 128.6 ($\underline{C}$quat.), 129.0 (Ar-$\underline{C}$H), 129.5 (Ar-$\underline{C}$H), 129.8 (Ar-$\underline{C}$H), 130.7 (Ar-$\underline{C}$H), 132.2 ($\underline{C}$quat.), 135.3 ($\underline{C}$quat.), 137.8 ($\underline{C}$quat.), 147.0 ($\underline{C}$quat.), 152.6 (Ar-$\underline{C}$H), 198.0 ($\underline{C}$quat.); ***m/z*** (EI) 271 ([M]$^{\bullet+}$, 100%), 256 ([M − CH_3]$^+$, 90%), 228 ([M − C(O)CH_3]$^+$, 40%).

Preparation and characterisation of 2-acetylbenz[*c*]acridine (2k’) and 2-acetyl-8-aza[4]helicene (2k)

Prepared by slight modification of the general procedure on 0.04 mmol of 3-(*p*-acetylstyryl)quinoline (solvent was dry degassed acetonitrile instead of toluene). The crude mixture was purified by column chromatography on silica gel eluting with pentane/ethyl acetate 1:1 to 1:4. The first isomer **2k’** was the colourless solid characterised below (4.4 mg, 41%). The second isomer **2k** was the colourless solid reported above (4.3 mg, 40%).

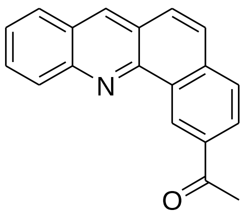

2k'

m.p. 150-152 °C; **FT-IR** ν_{max}(KBr) 2923, 1662, 1292, 852, 749 cm^{-1}; **^{1}H NMR** ($CDCl_3$, 600 MHz) δ_H 2.92 (s, 1H, C<u>*H*</u>$_3$), 7.65 (ddd, 1H, Ar-C<u>*H*</u>, *J* 8.0, 6.8, 1.1 Hz), 7.75 (d, 1H, Ar-C<u>*H*</u>, *J* 8.9 Hz), 7.88 (ddd, 1H, Ar-C<u>*H*</u>, *J* 8.4, 6.8, 1.6 Hz), 7.90 (d, 1H, Ar-C<u>*H*</u>, *J* 8.9 Hz), 7.95 (d, 1H, Ar-C<u>*H*</u>, *J* 8.2 Hz), 8.07-8.08 (m, 1H, Ar-C<u>*H*</u>), 8.34 (dd, 1H, Ar-C<u>*H*</u>, *J* 8.2, 1.9 Hz), 8.44 (d, 1H, Ar-C<u>*H*</u>, *J* 8.4 Hz), 8.72 (s, 1H, Ar-C<u>*H*</u>), 10.11 (s, 1H, Ar-C<u>*H*</u>); **^{13}C NMR** ($CDCl_3$, 150 MHz) δ_C 27.2 (<u>*C*</u>H$_3$), 125.3 (<u>*C*</u>quat.), 126.5 (Ar-<u>*C*</u>H), 126.8 (Ar-<u>*C*</u>H), 127.1 (Ar-<u>*C*</u>H), 127.3 (<u>*C*</u>quat.), 127.7 (Ar-<u>*C*</u>H), 128.1 (Ar-<u>*C*</u>H), 128.5 (Ar-<u>*C*</u>H), 128.7 (Ar-<u>*C*</u>H), 130.0 (Ar-<u>*C*</u>H), 130.4 (Ar-<u>*C*</u>H), 131.0 (<u>*C*</u>quat.), 131.4 (<u>*C*</u>quat.), 135.7 (Ar-<u>*C*</u>H), 137.2 (<u>*C*</u>quat.), 147.6 (<u>*C*</u>quat.), 148.1 (<u>*C*</u>quat.), 198.6 (<u>*C*</u>quat.); ***m/z*** (EI) 271 ($[M]^{\bullet+}$, 90%), 256 ($[M - CH_3]^+$, 95%), 228 ($[M - C(O)CH_3]^+$, 100%).

Preparation and characterisation of 2-bromo-6-methyl[4]helicene (2l)

Prepared according to the general procedure on 0.05 mmol of 1-(*p*-bromophenyl)-2-(2-naphthyl)propene. The crude mixture was purified by column chromatography on silica gel eluting with pentane to afford the title product as a colourless solid (12 mg, 75%).

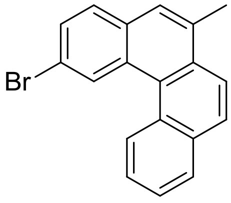

2l

m.p. 101-102 °C; **FT-IR** ν_{max}(KBr) 3049, 2100, 1587, 1484, 870, 790, 746 cm^{-1}; **^{1}H NMR** ($CDCl_3$, 600 MHz) δ_H 2.80 (d, 3H, C<u>*H*</u>$_3$, *J* 0.9 Hz), 7.64-7.67 (m, 1H, Ar-C<u>*H*</u>), 7.67 (dd, 1H, Ar-C<u>*H*</u>, *J* 8.5, 1.9 Hz), 7.69 (s, 1H, Ar-C<u>*H*</u>), 7.71-7.73 (m, 1H, Ar-C<u>*H*</u>), 7.79 (d, 1H, Ar-C<u>*H*</u>, *J* 8.5 Hz), 7.96 (d, 1H, Ar-C<u>*H*</u>, *J* 8.8 Hz), 8.02 (d, 1H, Ar-C<u>*H*</u>, *J* 8.8 Hz), 8.03-8.04 (m, 1H, Ar-C<u>*H*</u>), 9.01 (d, 1H, Ar-C<u>*H*</u>, *J* 8.4 Hz), 9.18 (s, 1H, Ar-C<u>*H*</u>); **^{13}C NMR** ($CDCl_3$, 150 MHz) δ_C 20.6 (<u>*C*</u>H$_3$), 119.6 (<u>*C*</u>quat.), 122.5 (Ar-<u>*C*</u>H), 126.2 (Ar-<u>*C*</u>H), 126.7 (Ar-<u>*C*</u>H), 126.9 (<u>*C*</u>quat.), 127.1 (Ar-<u>*C*</u>H), 127.9 (Ar-<u>*C*</u>H), 128.0 (Ar-<u>*C*</u>H), 128.5 (Ar-<u>*C*</u>H), 129.2 (Ar-<u>*C*</u>H), 129.3 (Ar-<u>*C*</u>H), 130.2 (<u>*C*</u>quat.), 130.4 (Ar-<u>*C*</u>H), 130.7 (<u>*C*</u>quat.), 131.2 (<u>*C*</u>quat.), 131.8 (<u>*C*</u>quat.), 133.1 (<u>*C*</u>quat.), 133.2 (<u>*C*</u>quat.); ***m/z*** (EI) 322 ($[M]^{\bullet+}$, 100%), 320 ($[M]^{\bullet+}$, 100%).

Preparation and characterisation of 2,6-dimethyl[4]helicene (2m)

Prepared according to the general procedure on 0.048 mmol of 1-(*p*-tolyl)-2-(2-naphthyl)propene. The crude mixture was purified by column chromatography on silica gel eluting with pentane to afford the title product as a colourless solid (9 mg, 73%).

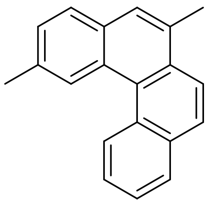

2m

m.p. 93-97 ºC; **FT-IR** ν_{max}(KBr) 3050 cm^{-1}, 2091 cm^{-1}, 1603 cm^{-1}, 1441 cm^{-1}, 878 cm^{-1}, 801 cm^{-1}, 747 cm^{-1}; **^{1}H NMR** ($CDCl_3$, 600 MHz) δ_H 2.63 (s, 3H, CH_3), 2.81 (s, 3H, CH_3), 7.43 (dd, 1H, Ar-C*H*, *J* 8.1, 1.2 Hz), 7.62 (m, 1H, Ar-C*H*), 7.67 (ddd, 1H, Ar-C*H*, *J* 8.5, 6.9, 1.5 Hz), 7.71 (s, 1H, Ar-C*H*), 7.84 (d, 1H, Ar-C*H*, *J* 8.0 Hz), 7.93 (d, 1H, Ar-C*H*, *J* 8.8 Hz), 8.03 (dd, 1H, Ar-C*H*, *J* 8.0, 1.3 Hz), 8.03 (d, 1H, Ar-C*H*, *J* 8.8 Hz), 8.85 (s, 1H, Ar-C*H*), 9.18 (d, 1H, Ar-C*H*, *J* 8.5 Hz); **^{13}C NMR** ($CDCl_3$, 150 MHz) δ_C 20.5 (CH_3), 22.3 (CH_3), 122.8 (Ar-CH), 125.8 (Ar-CH), 126.0 (Ar-CH), 127.2 (Ar-CH), 127.4 (Ar-CH), 127.6 (Cquat.), 127.7 (Ar-CH), 127.7 (Ar-CH), 127.9 (Ar-CH), 128.4 (Ar-CH), 128.5 (Ar-CH), 129.7 (Cquat.), 130.6 (Cquat.), 130.9 (Cquat.), 131.4 (Cquat.), 131.5 (Cquat.), 133.1 (Cquat.), 135.0 (Cquat.); ***m/z*** (EI) 256 ([M]$^{\bullet+}$, 100%).

Preparation and characterisation of 2-methoxy-6-methyl[4]helicene (2n)

Prepared according to the general procedure on 0.044 mmol of 1-(*p*-methoxyphenyl)-2-(2-naphthyl)propene. The crude mixture was purified by column chromatography on silica gel eluting with pentane to afford the title product as a colourless solid (12 mg, 99%).

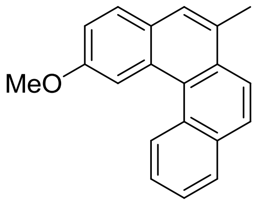

2n

m.p. 97-99 ºC; **FT-IR** ν_{max}(KBr) 2925 cm^{-1}, 1905 cm^{-1}, 1762 cm^{-1}, 1605 cm^{-1}, 1497 cm^{-1}, 1229 cm^{-1}, 1031 cm^{-1}, 805 cm^{-1}, 751 cm^{-1}; **^{1}H NMR** ($CDCl_3$, 600 MHz) δ_H 2.79 (d, 3H, CH_3, *J* 1.0 Hz), 4.01 (s, 3H, OCH_3), 7.27 (dd, 1H, Ar-C*H*, *J* 8.8, 2.6 Hz), 7.62 (ddd, 1H, Ar-C*H*, *J* 7.9, 6.9, 1.2 Hz), 7.66 (ddd, 1H, Ar-C*H*, *J* 8.5, 6.9, 1.6 Hz), 7.69 (s, 1H, Ar-C*H*), 7.86 (d, 1H, Ar-C*H*, *J* 8.7 Hz), 7.93 (d, 1H, Ar-C*H*, *J* 8.8 Hz), 8.03 (d, 2H, 2 × Ar-C*H*, *J* 8.8 Hz), 8.52 (d, 1H, Ar-C*H*, *J* 2.4 Hz), 9.18 (d, 1H, Ar-C*H*, *J* 8.3 Hz); **^{13}C NMR** ($CDCl_3$, 150 MHz) δ_C 20.4 (CH_3), 55.7 (OCH_3), 109.5 (Ar-CH), 116.6 (Ar-CH), 122.9 (Ar-CH), 125.8 (Ar-CH), 1260 (Ar-CH), 127.2 (Cquat.), 127.3 (Ar-CH), 127.4 (Ar-CH), 127.7 (Ar-CH), 128.4 (Cquat.), 128.5 (Ar-CH), 129.2 (Ar-CH), 130.0 (Cquat.), 130.6 (Cquat.), 130.7 (Cquat.), 131.3 (Cquat.), 133.0 (Cquat.), 157.7 (Cquat.); ***m/z*** (EI) 272 ([M]$^{\bullet+}$, 50%), 227 ([M – OC_2H_6]$^+$, 95%).

Preparation and characterisation of 2-bromo-6-methyl-9,10-dimethoxy[5]helicene (2o)

Prepared according to the general procedure on 0.064 mmol of the corresponding olefin. The crude mixture was purified by column chromatography on silica gel eluting with pentane/diethyl ether 20:1 to afford the title product as a colourless foam (18 mg, 65%).

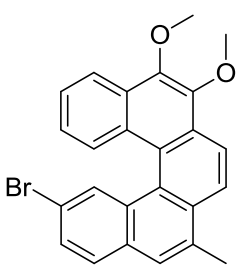

2o

FT-IR ν_{max}(KBr) 2928 cm^{-1}, 1600 cm^{-1}, 1094 cm^{-1}, 1054 cm^{-1}, 784 cm^{-1}; **^{1}H NMR** (CDCl$_3$, 600 MHz) δ_H 2.8 (s, 3H, C$\underline{H}_3$), 4.15 (s, 3H, OC$\underline{H}_3$), 4.22 (s, 3H, OC$\underline{H}_3$), 7.27 (ddd, 1H, Ar-C$\underline{H}_{13}$, *J* 8.6, 6.8, 1.5 Hz), 7.53 (dd, 1H, Ar-C$\underline{H}_3$, *J* 8.5, 1.9 Hz), 7.56 (ddd, 1H, Ar-C$\underline{H}_{12}$, J 8.0, 6.8, 1.0 Hz), 7.68 (s, 1H, Ar-C$\underline{H}_5$), 7.70 (d, 1H, Ar-C$\underline{H}_4$, *J* 8.5 Hz), 8.08 (d, 1H, Ar-C$\underline{H}_7$, *J* 8.7 Hz), 8.29-8.31 (m, 1H, Ar-C$\underline{H}_{11}$) 8.31 (d, 1H, Ar-C$\underline{H}_8$, *J* 8.7 Hz), 8.39 (d, 1H, Ar-C$\underline{H}_{14}$, *J* 8.6 Hz), 8.53 (d, 1H, Ar-C$\underline{H}_1$, *J* 1.9 Hz); **^{13}C NMR** (CDCl$_3$, 125 MHz) δ_C 20.3 ($\underline{C}$H$_3$), 61.1 (O$\underline{C}$H$_3$), 61.3 (O$\underline{C}$H$_3$), 120.5 ($\underline{C}$quat.), 121.3 (Ar-$\underline{C}$H), 121.6 (Ar-$\underline{C}$H), 123.2 (Ar-$\underline{C}$H), 123.8 (Ar-$\underline{C}$H), 124.8 ($\underline{C}$quat.), 126.3 ($\underline{C}$quat.), 126.5 (Ar-$\underline{C}$H), 126.7 (Ar-$\underline{C}$H), 128.6 (Ar-$\underline{C}$H), 128.6 ($\underline{C}$quat.), 128.9 (Ar-$\underline{C}$H), 128.9 ($\underline{C}$quat.), 129.1 ($\underline{C}$quat.), 129.3 (Ar-$\underline{C}$H), 130.7 ($\underline{C}$quat.), 131.3 ($\underline{C}$quat.), 131.3 (Ar-$\underline{C}$H), 132.0 ($\underline{C}$quat.), 132.5 ($\underline{C}$quat.), 143.3 ($\underline{C}$quat.), 144.7 ($\underline{C}$quat.); ***m/z*** (EI) 432 ([M]$^{\bullet+}$, 100%), 430 ([M]$^{\bullet+}$, 95%).

Optimisation of the scale-up

Entry	*Retention time (min)*	*Scale (mmol)*	*Concentration (mmol.L^{-1})*	*Isolated yield (%)*
1	120	0.064	2	65
2	60	0.038	2	85
3	30	0.04	2	87
4	30	0.046	4	50
**5**	**24**	**0.06**	**2**	**93**
**6**	**24**	**0.36**	**2**	**85**
7	18	0.047	2	73
8	12	0.053	2	66

Preparation and characterisation of 3,4-dimethoxy-8,12-dimethylbenzo[*ghi*]perylene (2p)

Prepared according to the general procedure on 0.057 mmol of the corresponding olefin. The crude mixture was purified by column chromatography on silica gel eluting with pentane/diethyl ether 20:1 to afford the title product as a yellow foam (12 mg, 58%).

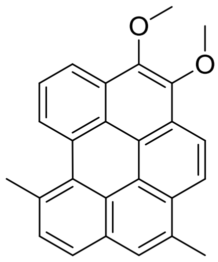

2p

FT-IR ν_{max}(KBr) 2928 cm^{-1}, 1620 cm^{-1}, 1341 cm^{-1}, 1097 cm^{-1}, 1008 cm^{-1}, 796 cm^{-1}, 741 cm^{-1}; ^{1}**H NMR** ($CDCl_3$, 600 MHz) δ_H 2.97 (s, 3H, C*H*$_3$), 3.32 (s, 3H, C*H*$_3$), 4.28 (s, 3H, OC*H*$_3$), 4.29 (s, 3H, OC*H*$_3$), 7.89 (d, 1H, Ar-C*H$_2$*, *J* 7.9 Hz), 7.94 (s, 1H, Ar-C*H$_4$*), 8.05 (d, 1H, Ar-C*H$_3$*, *J* 7.9 Hz), 8.08 (t, 1H, Ar-C*H$_{11}$*, J 7.9 Hz), 8.58 (d, 1H, Ar-C*H$_7$*, *J* 8.6 Hz), 8.60 (d, 1H, Ar-C*H$_{10}$*, *J* 7.5 Hz), 8.79 (d, 1H, Ar-C*H$_6$*, J 8.6 Hz), 8.96 (d, 1H, Ar-C*H$_{12}$*, *J* 7.9 Hz); ^{13}**C NMR** ($CDCl_3$, 125 MHz) δ_C 20.3 (*C*H$_3$), 27.8 (*C*H$_3$), 61.4 (O*C*H$_3$), 61.5 (O*C*H$_3$), 119.4 (Ar-*C*H), 119.9 (Ar-*C*H), 122.3 (*C*quat.), 122.4 (Ar-*C*H), 124.2 (*C*quat.), 125.1 (*C*quat.), 125.3 (*C*quat.), 125.4 (Ar-*C*H), 125.7 (Ar-*C*H), 125.8 (Ar-*C*H), 126.0 (*C*quat.), 127.1 (Ar-*C*H), 128.8 (*C*quat.), 129.0 (*C*quat.), 131.3 (*C*quat.), 131.9 (Ar-*C*H), 132.3 (*C*quat.), 133.1 (*C*quat.), 144.6 (*C*quat.), 144.7 (*C*quat.); ***m/z*** (EI) 364 ([M]$^{\bullet+}$, 100%), 349 ([M – CH$_3$]$^+$, 40%).

Preparation and characterisation of 3,4,8-trimethoxy-12-methylbenzo[*ghi*]perylene (2q)

Prepared according to the general procedure on 0.101 mmol of the corresponding olefin. The crude mixture was purified by column chromatography on silica gel eluting with pentane/diethyl ether 10:1 to afford the title product as a colourless foam (20 mg, 52%).

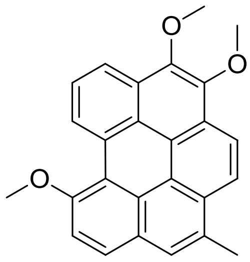

2q

FT-IR ν_{max}(KBr) 2930 cm^{-1}, 1616 cm^{-1}, 1449 cm^{-1}, 1336 cm^{-1}, 1244 cm^{-1}, 1096 cm^{-1}, 989 cm^{-1}, 801 cm^{-1}, 748 cm^{-1}; ^{1}**H NMR** ($CDCl_3$, 600 MHz) δ_H 2.93 (s, 3H, C*H*$_3$), 4.28 (s, 3H, OC*H*$_3$), 4.29 (s, 3H, OC*H*$_3$), 4.30 (s, 3H, OC*H*$_3$), 7.72 (d, 1H, Ar-C*H$_2$*, *J* 7.7 Hz), 7.87 (s, 1H, Ar-C*H$_4$*), 8.11 (m, 2H, 2 × Ar-C*H*), 8.54 (t, 1H, Ar-C*H$_{11}$*, J 8.6 Hz), 8.59 (dd, 1H, Ar-C*H$_7$*, *J* 7.9, 1.0 Hz), 8.79 (d, 1H, Ar-C*H$_{10}$*, *J* 8.6 Hz), 10.01 (dd, 1H, Ar-C*H$_6$*, J 8.1, 1.0 Hz); ^{13}**C NMR** ($CDCl_3$, 125 MHz) δ_C 20.2 (*C*H$_3$), 56.5 (O*C*H$_3$), 61.3 (O*C*H$_3$), 61.4 (O*C*H$_3$), 112.2 (Ar-*C*H), 117.9 (*C*quat.), 119.6 (Ar-*C*H), 119.7 (Ar-*C*H), 122.0 (Ar-*C*H), 122.7 (*C*quat.), 124.3 (*C*quat.), 124.7 (*C*quat.), 125.0 (*C*quat.), 125.9 (Ar-*C*H), 126.4 (*C*quat.), 126.5 (Ar-*C*H), 126.7 (*C*quat.), 127.0 (Ar-*C*H), 127.0 (Ar-*C*H), 128.7 (*C*quat.), 129.1 (*C*quat.), 130.1 (*C*quat.), 130.7 (*C*quat.), 144.4 (*C*quat.), 144.6 (*C*quat.), 156.6 (*C*quat.); ***m/z*** (EI) 380 ([M]$^{\bullet+}$, 100%), 365 ([M – CH$_3$]$^+$, 50%).

Preparation and characterisation of the 1:1 mixture of 5,6-dimethoxy-9-methyl[6]helicene (2r) and its ribbon-like regioisomer (2r')

Prepared according to the general procedure on 0.05 mmol of the corresponding olefin. The crude mixture was purified by column chromatography on silica gel eluting with pentane/diethyl ether 20:1 to afford the inseparable mixture of the title products as a yellow oil (15 mg, 75%).

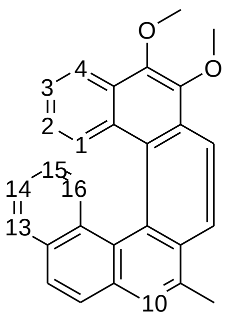

2r

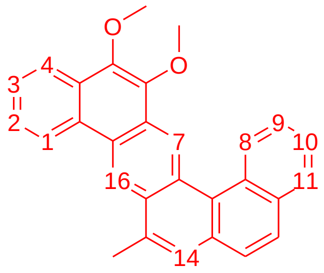

2r'

^{1}H NMR ($CDCl_3$, 600 MHz) δ_H 2.91 (d, 3H, C$\underline{H}_3$, *J* 0.8 Hz), 3.01 (d, 3H, C$\underline{H}_3$, *J* 0.9 Hz), 4.20, 4.21, 4.22, 4.26 (s, 4 × 3H, 4 × OC$\underline{H}_3$), 6.62 (ddd, 1H, Ar-C$\underline{H}_2$, *J* 8.4, 6.8, 1.3 Hz), 6.68 (ddd, 1H, Ar-C$\underline{H}_{15}$, *J* 8.4, 6.9, 1.4 Hz), 7.17 (ddd, 1H, Ar-C$\underline{H}_{14}$, *J* 7.9, 6.9, 1.1 Hz), 7.22 (ddd, 1H, Ar-C$\underline{H}_3$, *J* 8.0, 6.8, 1.1 Hz), 7.48 (dd, 1H, Ar-C$\underline{H}_{16}$, *J* 8.4 Hz), 7.54 (dd, 1H, Ar-C$\underline{H}_1$, *J* 8.4 Hz), 7.65 (ddd, 1H, Ar-C$\underline{H}_{10}$, *J* 0.9, 6.9, 7.8 Hz), 7.70 (s, 1H, Ar-C$\underline{H}_{14}$), 7.71-7.72 (m, 2H, Ar-C$\underline{H}_2$ and Ar-C$\underline{H}_3$), 7.77-7.79 (m, 2H, Ar-C$\underline{H}_9$ and Ar-C$\underline{H}_{10}$), 7.80 (s, 1H, Ar-C$\underline{H}_{10}$), 7.82 (d, 1H, Ar-C$\underline{H}$, *J* 8.4 Hz), 7.87 (AA' system, 2H, 2 × Ar-C$\underline{H}$), 7.96 (d, 1H, Ar-C$\underline{H}$, *J* 8.4 Hz), 8.06 (dd, 1H, Ar-C$\underline{H}_{11}$, *J* 7.8, 0.8 Hz), 8.16 (dd, 1H, Ar-C$\underline{H}_4$, *J* 8.0, 1.1 Hz), 8.23 (d, 1H, Ar-C$\underline{H}$, *J* 8.7 Hz), 8.29-8.31 (m, 1H, Ar-C$\underline{H}_4$), 8.41 (d, 1H, Ar-C$\underline{H}$, *J* 8.7 Hz), 8.98-8.91 (m, 1H, Ar-C$\underline{H}_1$), 9.34 (d, 1H, Ar-C$\underline{H}_8$, *J* 8.5 Hz), 9.38 (s, 1H, Ar-C$\underline{H}_{16}$), 9.99 (s, 1H, Ar-C$\underline{H}_7$);

Differential NOESY irradiating proton 7 showed enhancement of the signal of 8. Differential NOESY irradiating proton 16 showed enhancement of the signals of 1 and CH_3.

^{13}C NMR ($CDCl_3$, 125 MHz) δ_C 20.2 ($\underline{C}H_3$), 20.2 ($\underline{C}H_3$), 61.2 (O$\underline{C}H_3$), 61.3 (O$\underline{C}H_3$), 61.4 (O$\underline{C}H_3$), 61.5 (O$\underline{C}H_3$), 118.2 (Ar-$\underline{C}$H), 120.6 (Ar-$\underline{C}$H), 121.1 (Ar-$\underline{C}$H), 121.9 (Ar-$\underline{C}$H), 122.5 (Ar-$\underline{C}$H), 123.1 (Ar-$\underline{C}$H), 123.2 (Ar-$\underline{C}$H), 124.0 (Ar-$\underline{C}$H), 124.7 ($\underline{C}$quat.), 124.8 (Ar-$\underline{C}$H), 125.1 (Ar-$\underline{C}$H), 125.6 (Ar-$\underline{C}$H), 125.7 (Ar-$\underline{C}$H), 126.0 (Ar-$\underline{C}$H), 126.2 (Ar-$\underline{C}$H), 126.5 ($\underline{C}$quat.), 126.6 (Ar-$\underline{C}$H), 126.6 (Ar-$\underline{C}$H), 126.9 ($\underline{C}$quat.), 127.0 ($\underline{C}$quat.), 127.2 ($\underline{C}$quat.), 127.4 (2 × Ar-$\underline{C}$H), 127.5 (Ar-$\underline{C}$H), 127.6 (Ar-$\underline{C}$H), 127.6 (Ar-$\underline{C}$H), 127.8 (Ar-$\underline{C}$H), 128.0 ($\underline{C}$quat.), 128.0 (Ar-$\underline{C}$H), 128.1 ($\underline{C}$quat.), 128.1 (Ar-$\underline{C}$H), 128.1 (Ar-$\underline{C}$H), 128.6 ($\underline{C}$quat.), 128.8 (Ar-$\underline{C}$H), 129.2 ($\underline{C}$quat.), 129.5 ($\underline{C}$quat.), 129.8 ($\underline{C}$quat.), 130.0 ($\underline{C}$quat.), 130.0 ($\underline{C}$quat.), 130.7 ($\underline{C}$quat.), 131.0 ($\underline{C}$quat.), 131.1 ($\underline{C}$quat.), 131.4 ($\underline{C}$quat.), 131.6 ($\underline{C}$quat.), 132.4 ($\underline{C}$quat.), 132.8 ($\underline{C}$quat.), 133.4 ($\underline{C}$quat.), 133.5 ($\underline{C}$quat.), 143.4 ($\underline{C}$quat.), 144.0 (2 × $\underline{C}$quat.), 144.9 ($\underline{C}$quat.).

4. ^{1}H and ^{13}C NMR spectra of new compounds

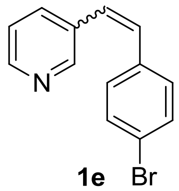

1e

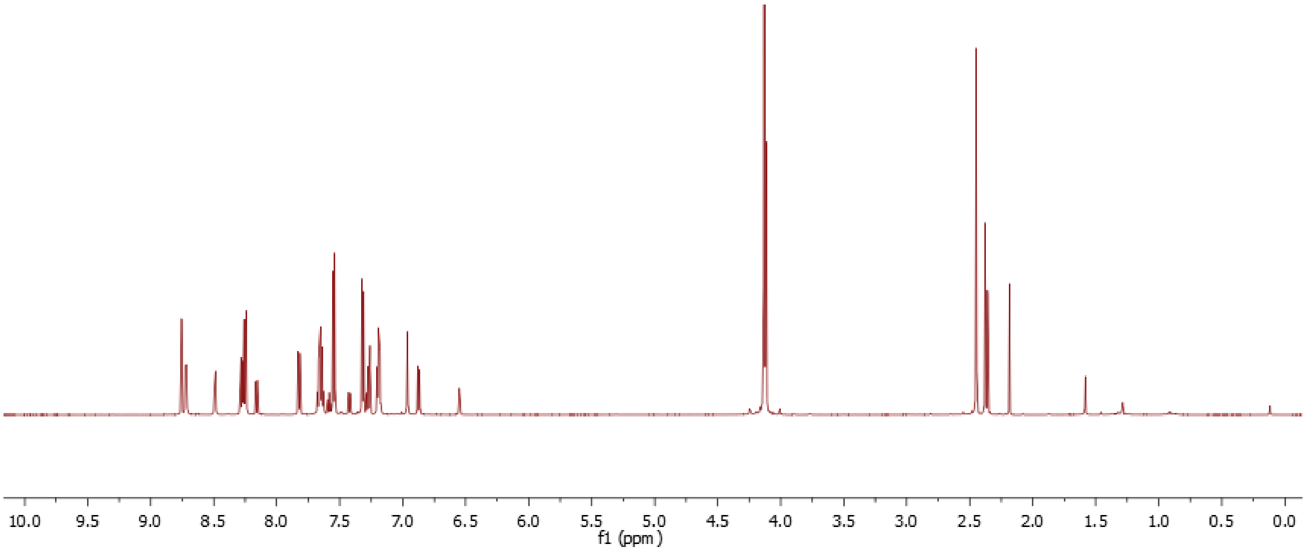

^{1}H NMR spectrum of 3-(*p*-bromostyryl)pyridine (1e)

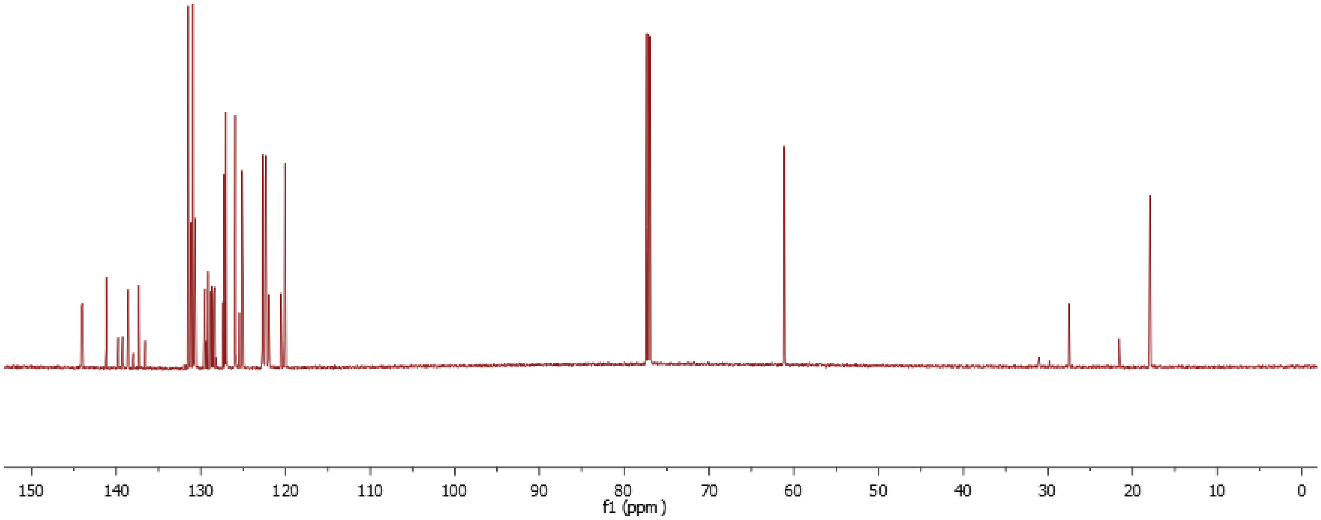

^{13}C NMR spectrum of 3-(*p*-bromostyryl)pyridine (1e)

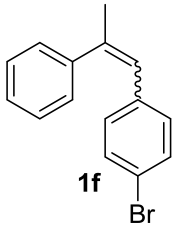

1f

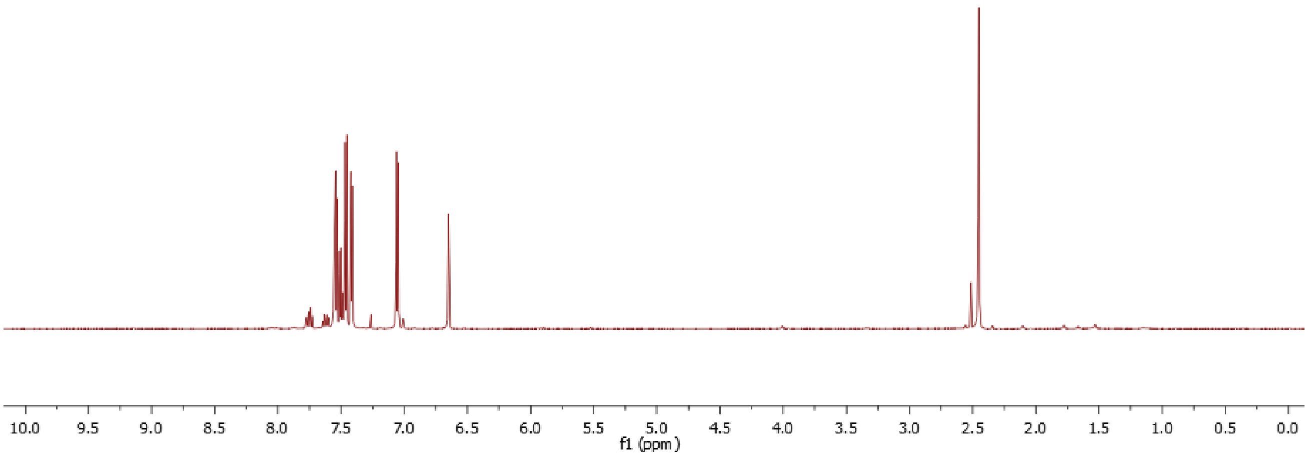

^{1}H NMR spectrum of 1-(*p*-bromophenyl)-2-phenylpropene (1f)

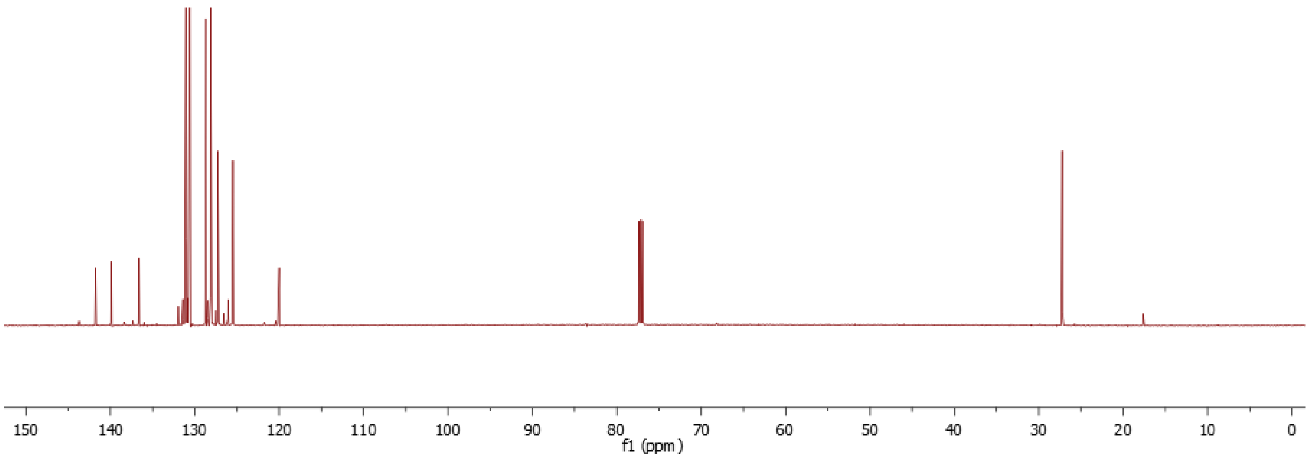

^{13}C NMR spectrum of 1-(*p*-bromophenyl)-2-phenylpropene (1f)

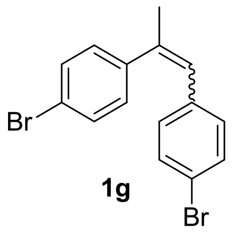

1g

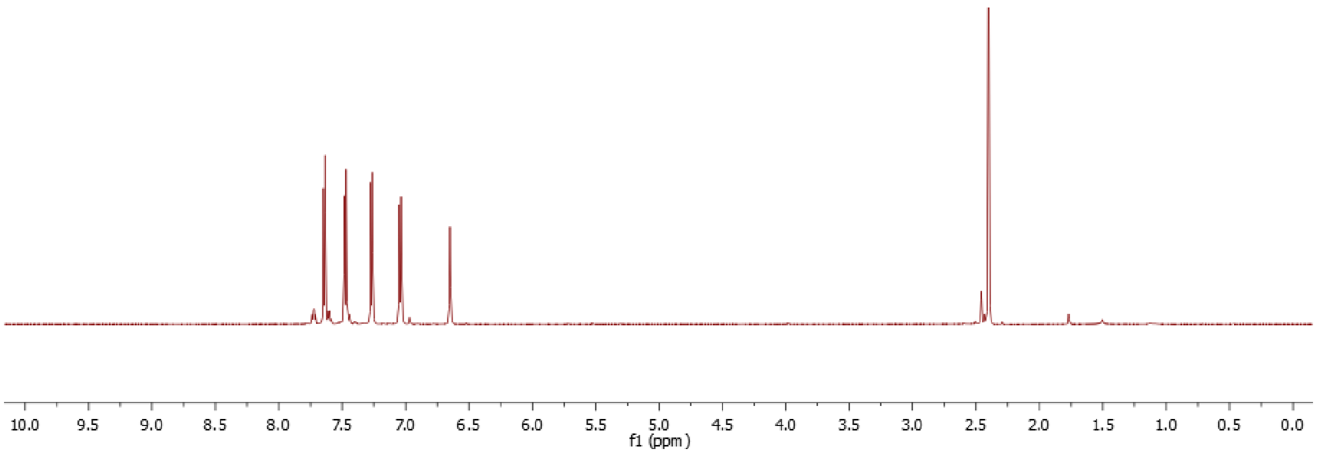

^{1}H NMR spectrum of 1,2-bis(*p*-bromophenyl)propene (1g)

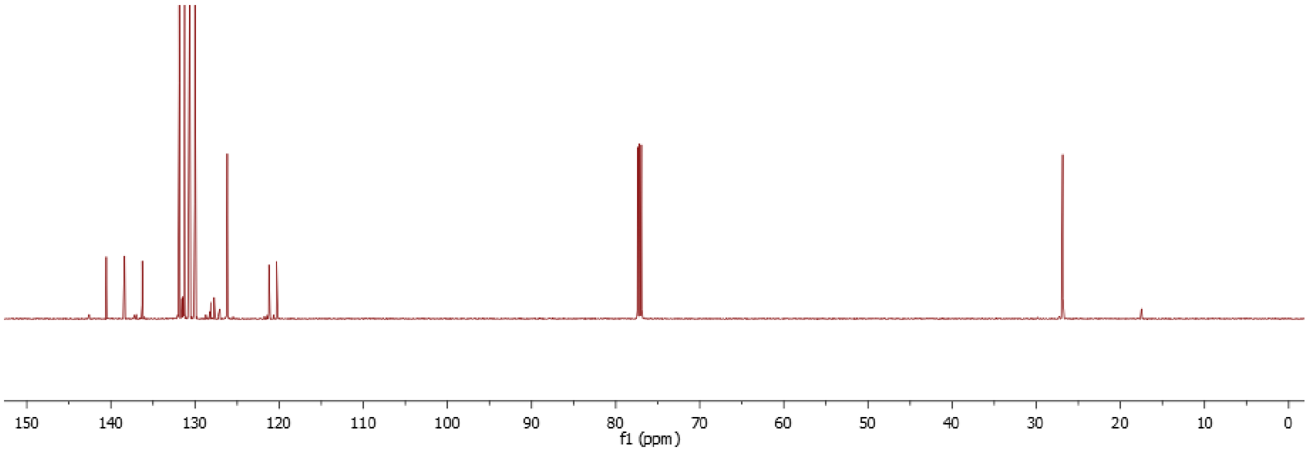

^{13}C NMR spectrum of 1,2-bis(*p*-bromophenyl)propene (1g)

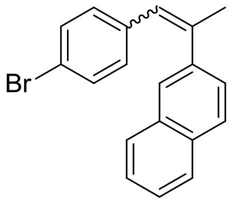

1l

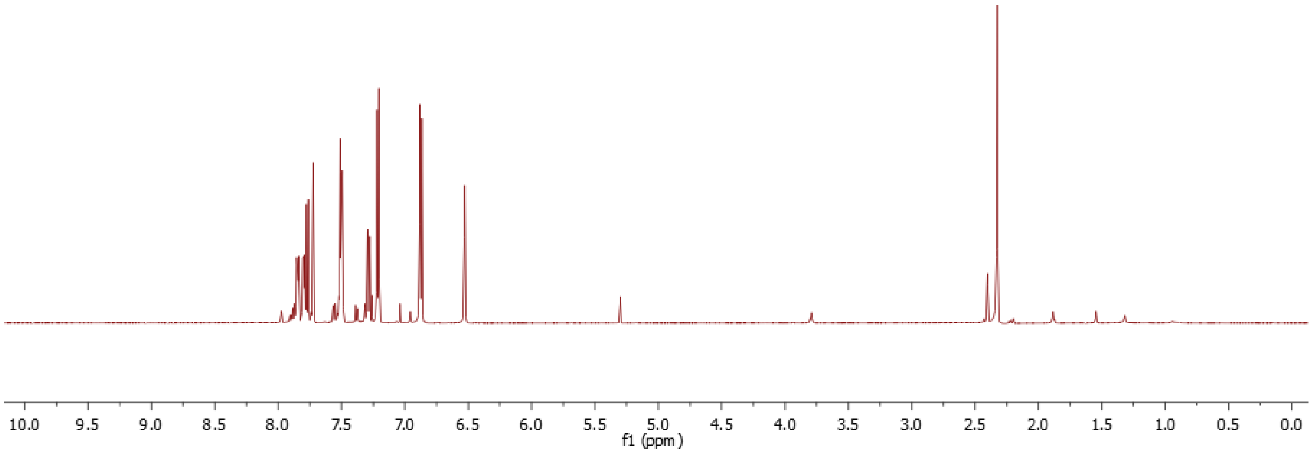

^{1}H NMR spectrum of 1-(*p*-bromophenyl)-2-(2-naphthyl)propene (1l)

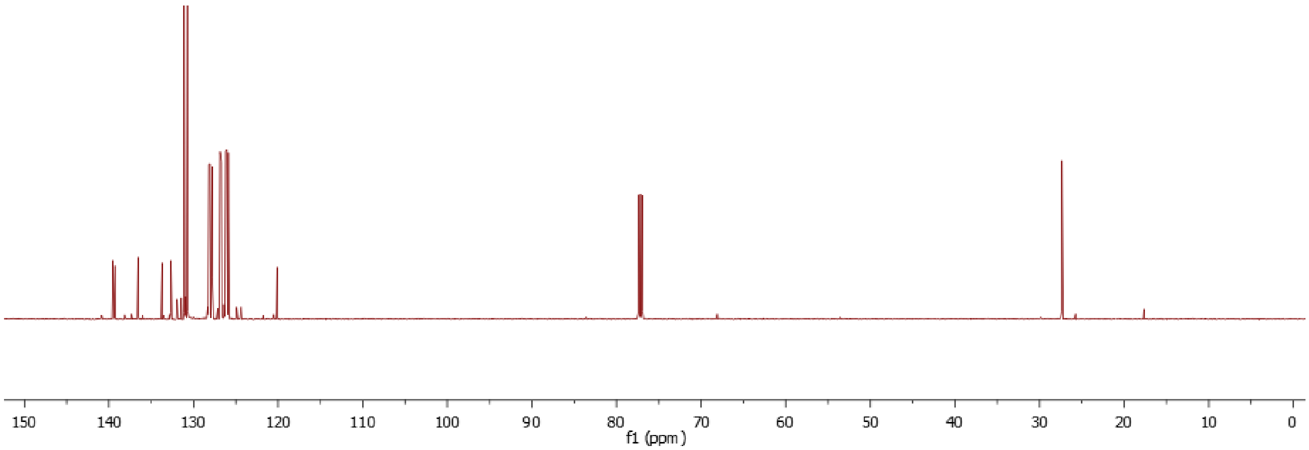

^{13}C NMR spectrum of 1-(*p*-bromophenyl)-2-(2-naphthyl)propene (1l)

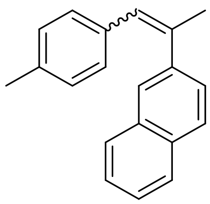

1m

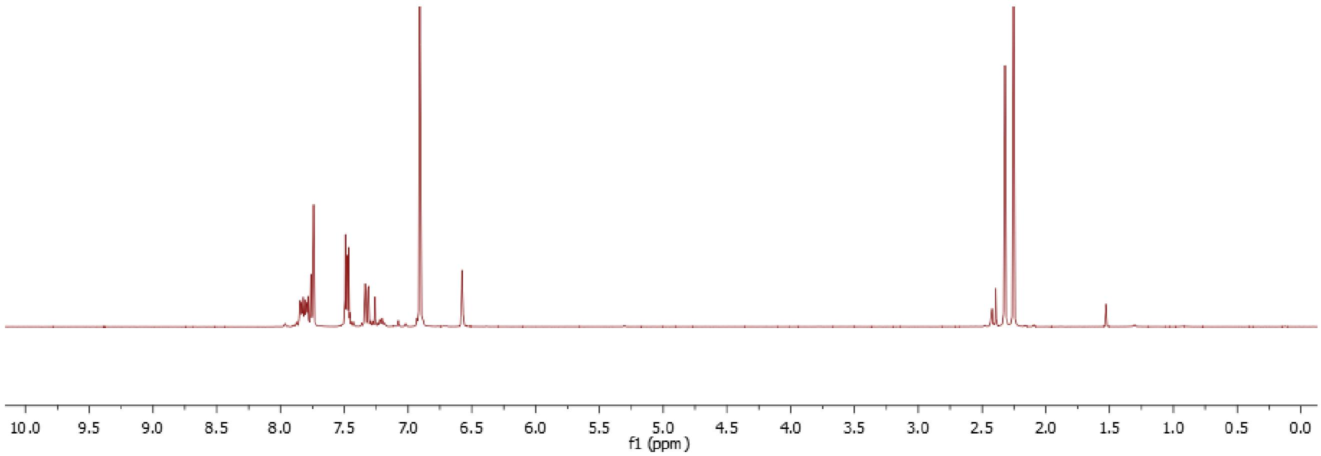

^{1}H NMR spectrum of 1-(*p*-tolyl)-2-(2-naphthyl)propene (1m)

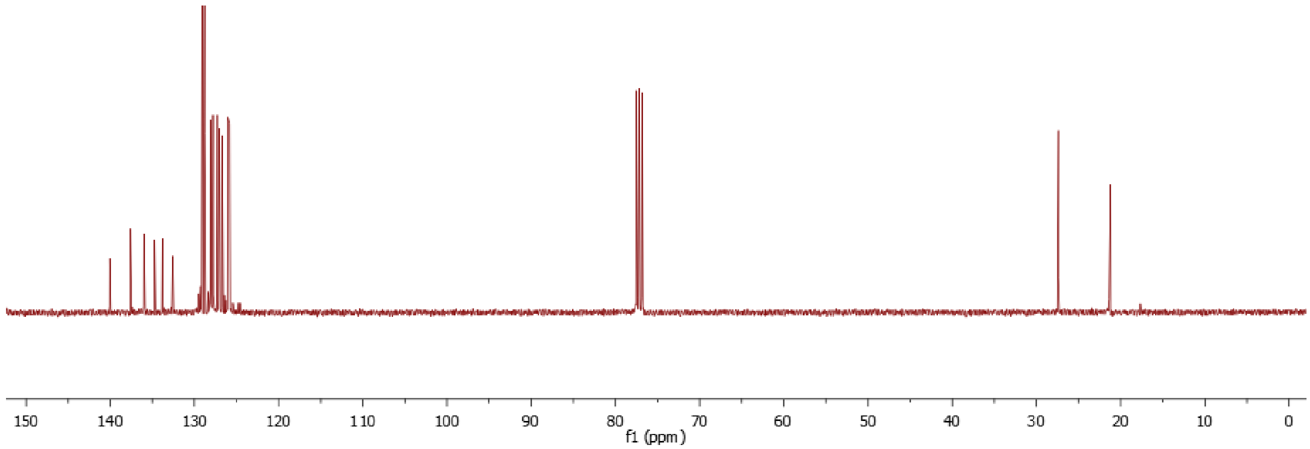

^{13}C NMR spectrum of 1-(*p*-tolyl)-2-(2-naphthyl)propene (1m)

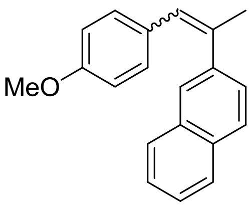

1n

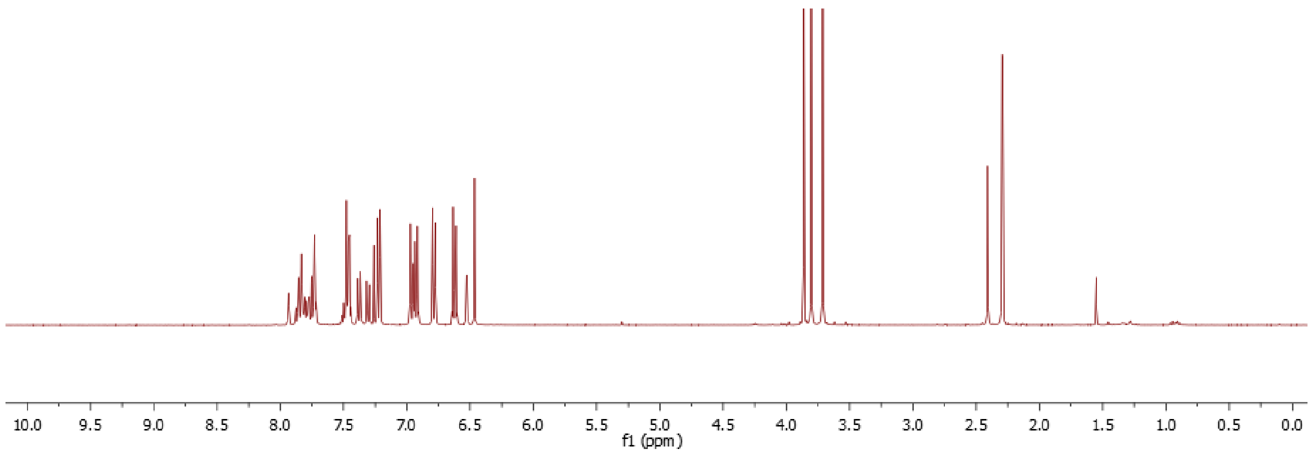

^{1}H NMR spectrum of 1-(*p*-methoxyphenyl)-2-(2-naphthyl)propene (1n)

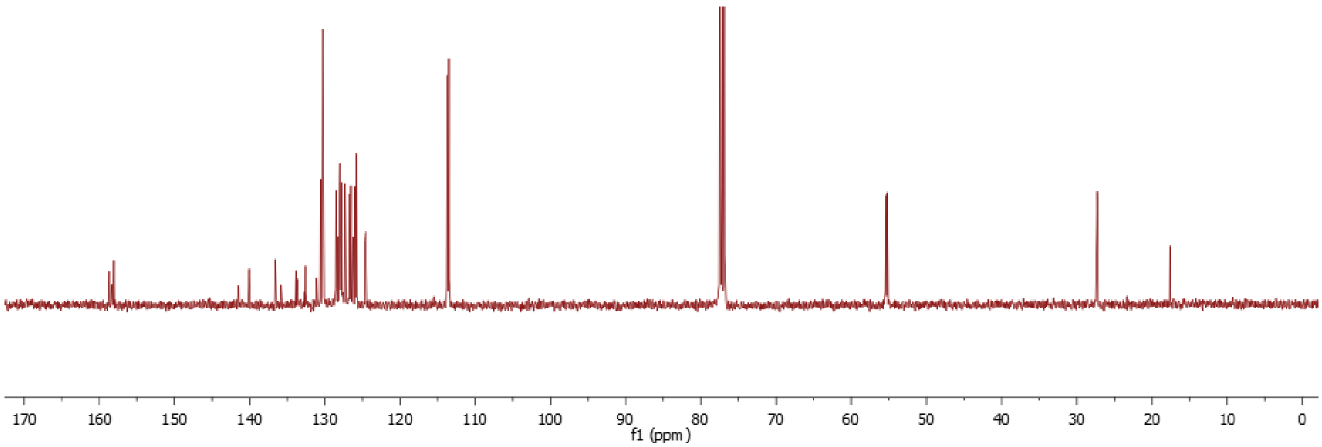

^{13}C NMR spectrum of 1-(*p*-methoxyphenyl)-2-(2-naphthyl)propene (1n)

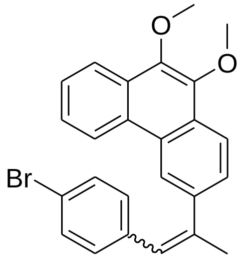

1o

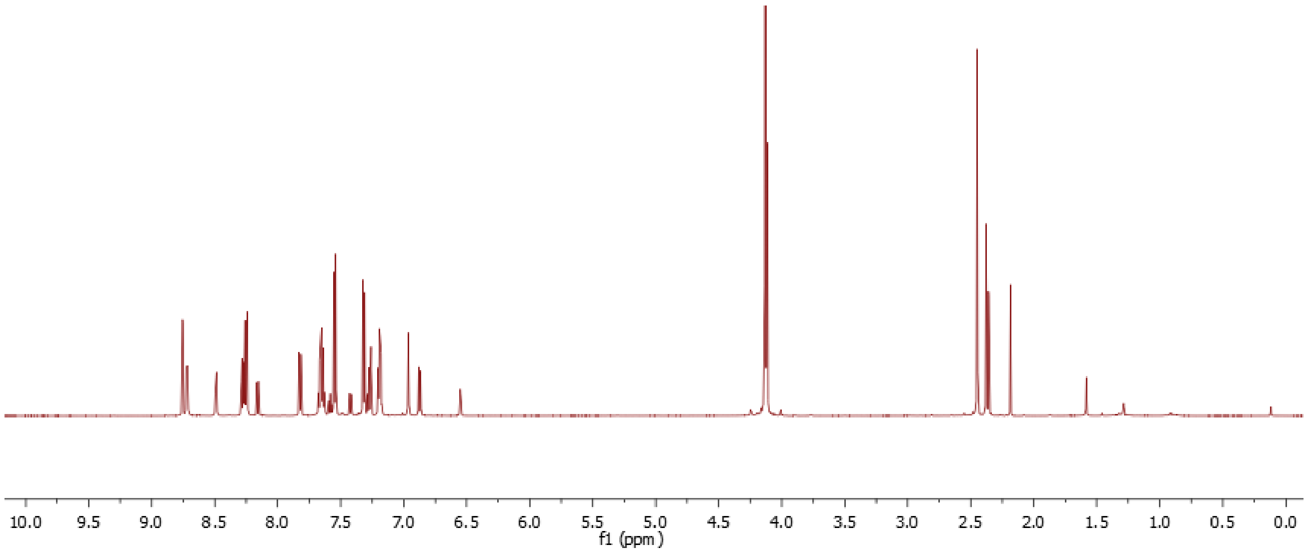

^{1}H NMR spectrum of 1-(*p*-bromophenyl)-2-[3-(9,10-dimethoxyphenanthrene)]propene (1o)

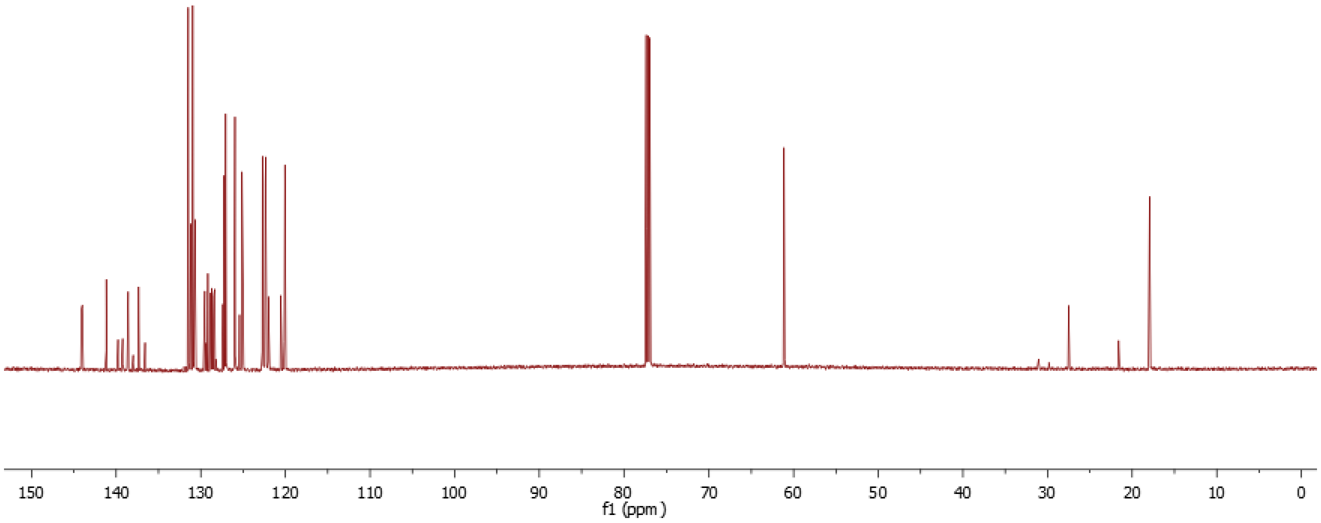

^{13}C NMR spectrum of 1-(*p*-bromophenyl)-2-[3-(9,10-dimethoxyphenanthrene)]propene (1o)

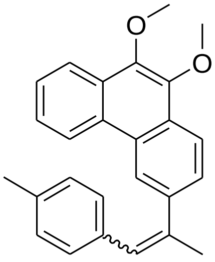

1p

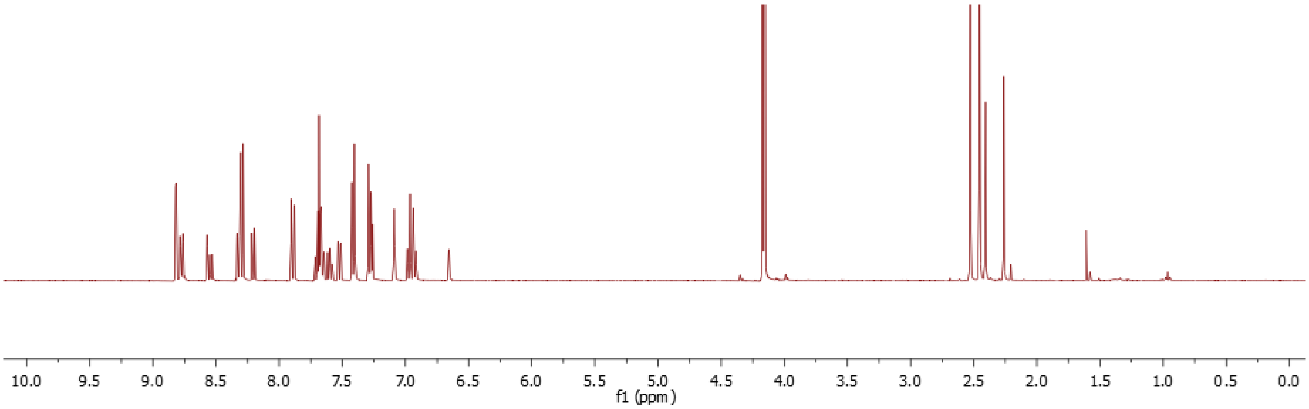

^{1}H NMR spectrum of 1-(*p*-tolyl)-2-[3-(9,10-dimethoxyphenanthrene)]propene (1p)

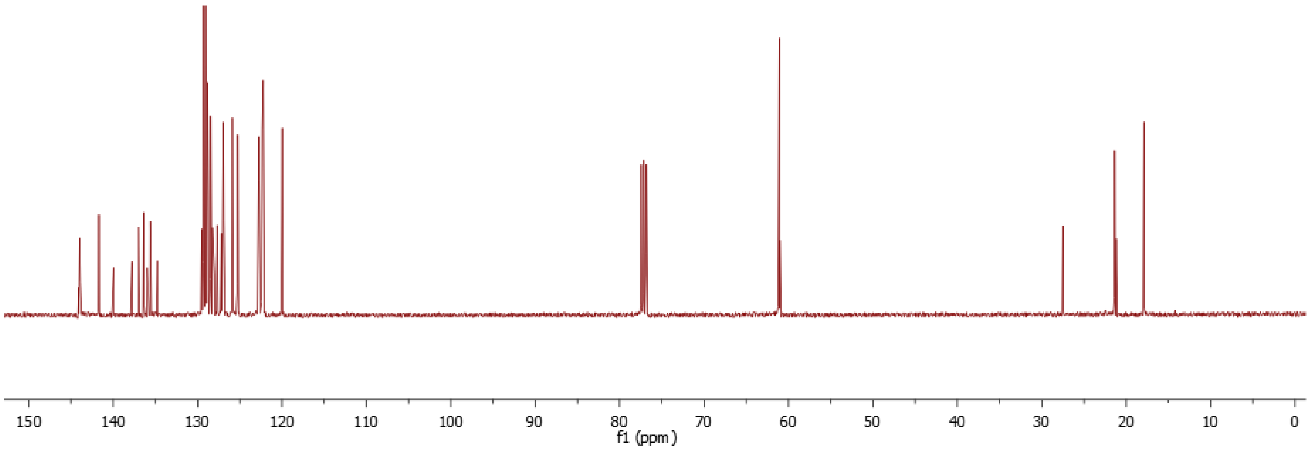

^{13}C NMR spectrum of 1-(*p*-tolyl)-2-[3-(9,10-dimethoxyphenanthrene)]propene (1p)

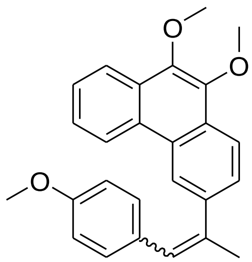

1q

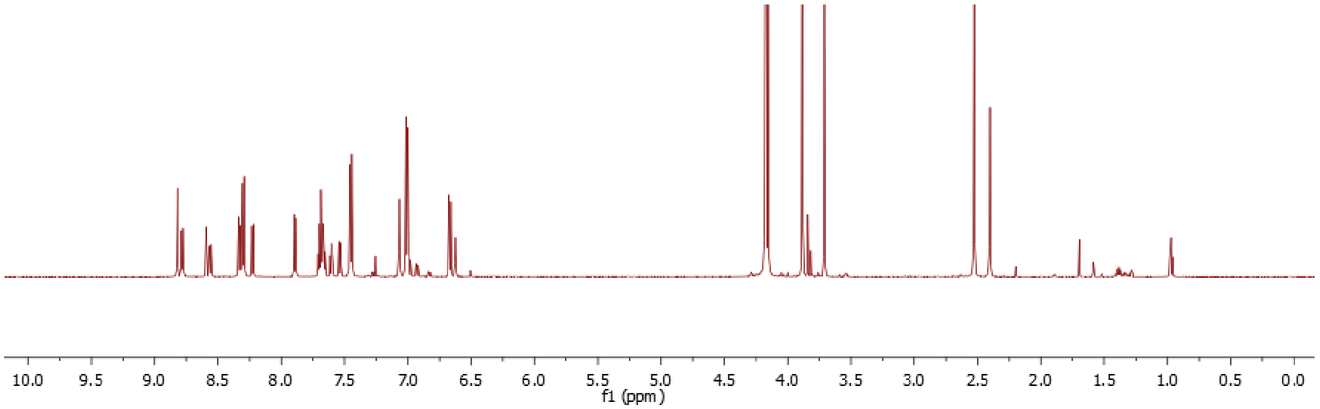

^{1}H NMR spectrum of 1-(*p*-methoxyphenyl)-2-[3-(9,10-dimethoxyphenanthrene)]propene (1q)

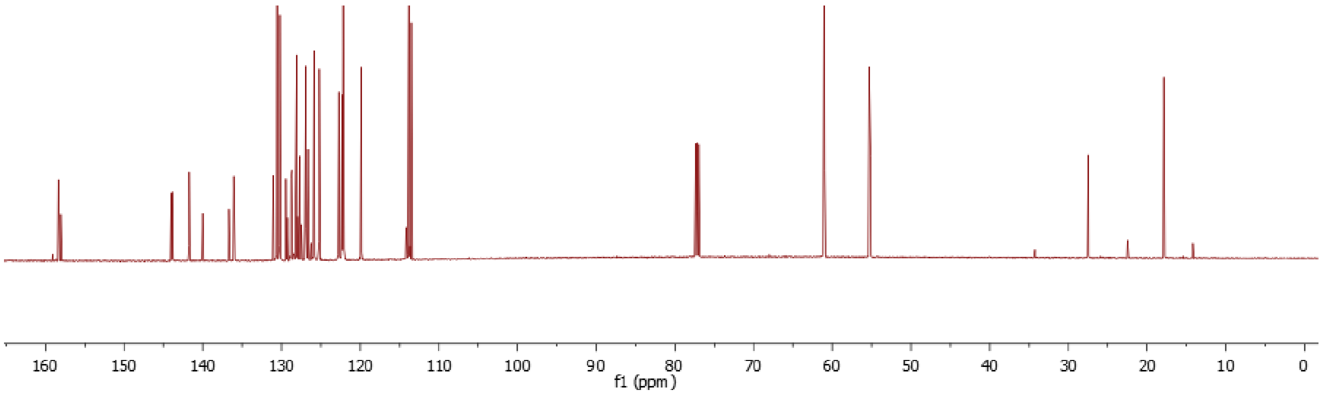

^{13}C NMR spectrum of 1-(*p*-methoxyphenyl)-2-[3-(9,10-dimethoxyphenanthrene)]propene (1q)

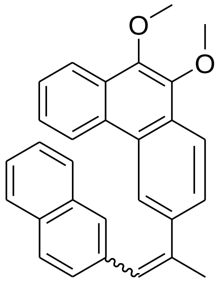

1r

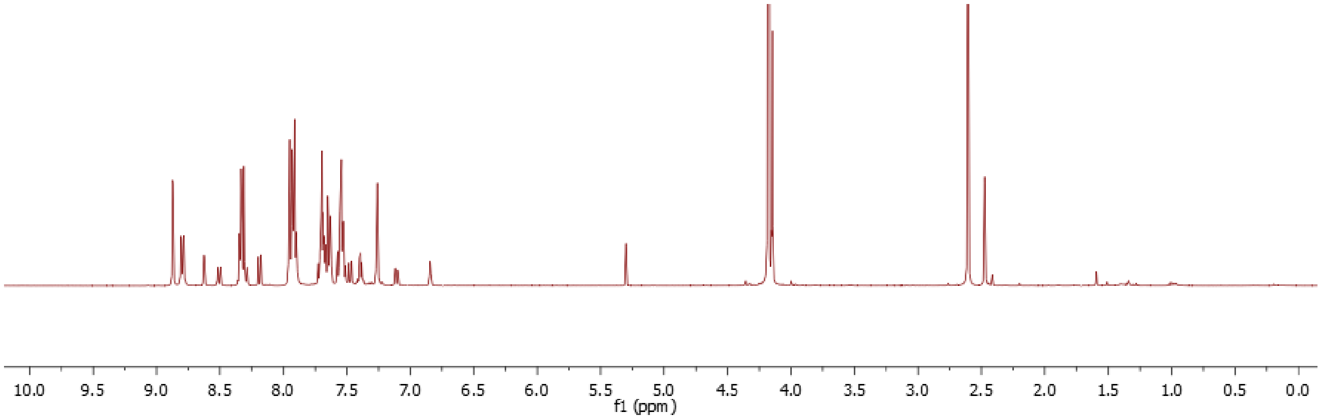

^{1}H NMR spectrum of 1-(2-naphtyl)-2-[3-(9,10-dimethoxyphenanthrene)]propene (1r)

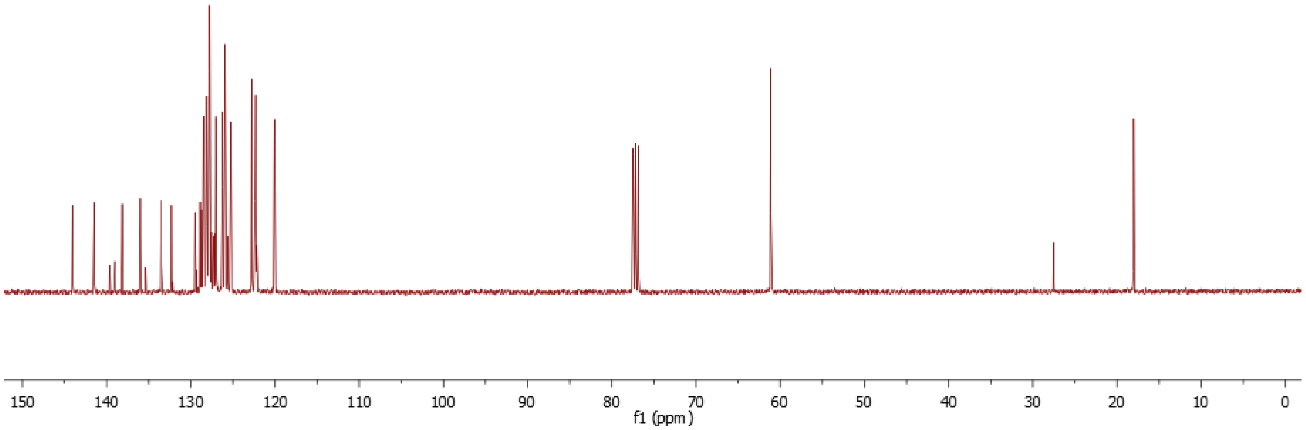

^{13}C NMR spectrum of 1-(2-naphtyl)-2-[3-(9,10-dimethoxyphenanthrene)]propene (1r)

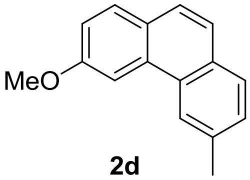

2d

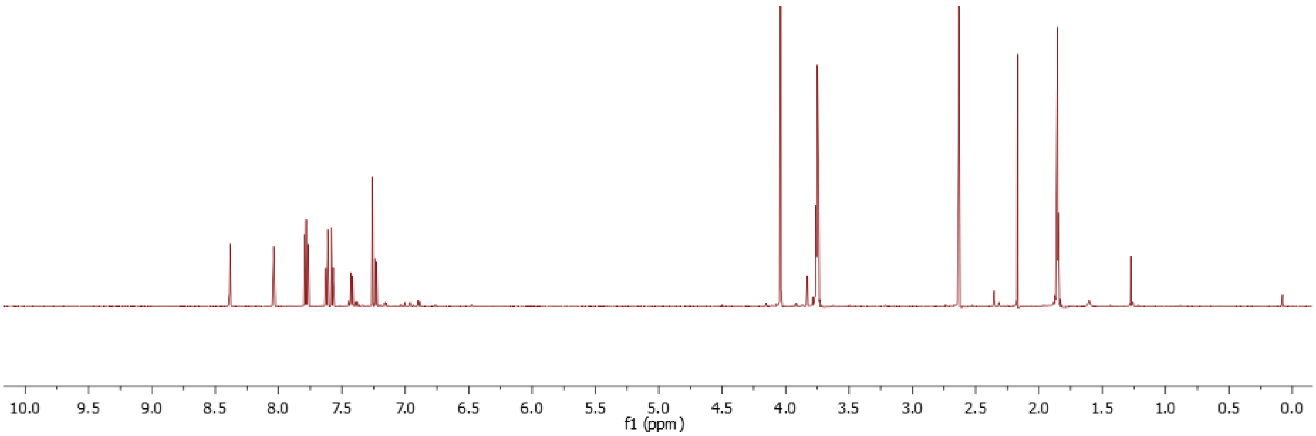

^{1}H NMR spectrum of 3-methoxy-6-methylphenanthrene (2d)

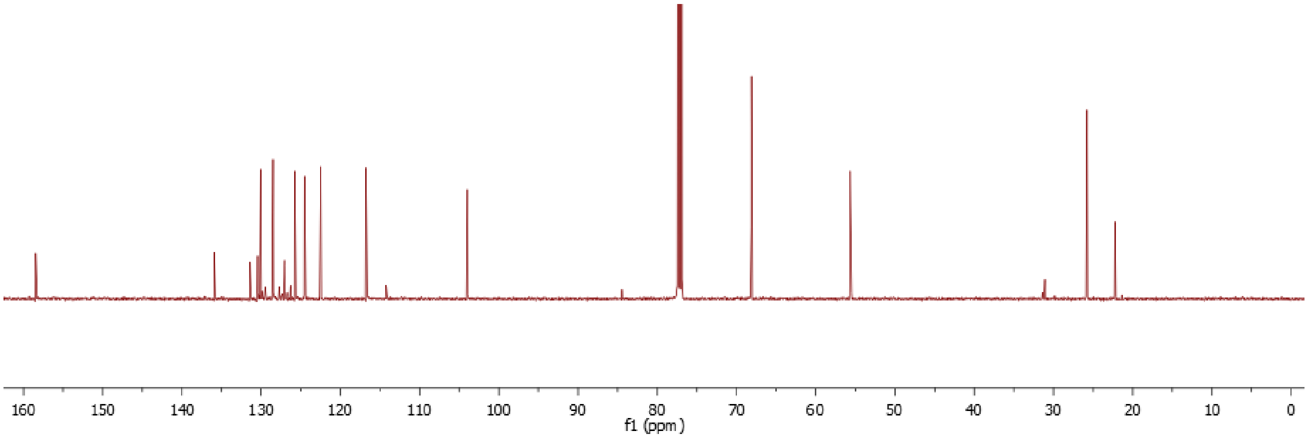

^{13}C NMR spectrum of 3-methoxy-6-methylphenanthrene (2d)

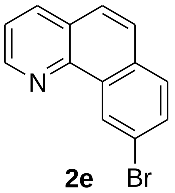

2e

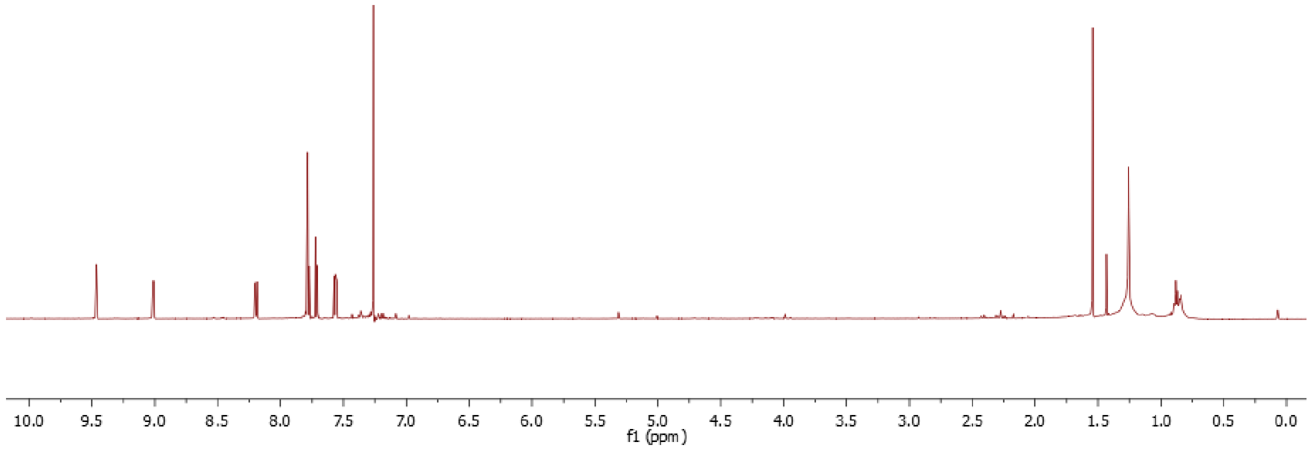

^{1}H NMR spectrum of 6-bromo-4-azaphenanthrene (2e)

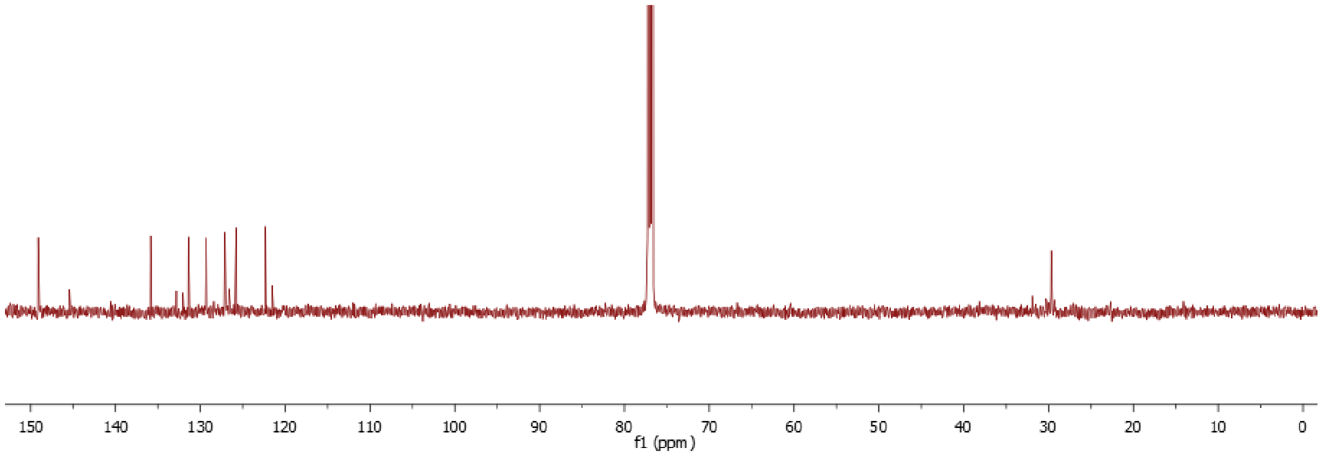

^{13}C NMR spectrum of 6-bromo-4-azaphenanthrene (2e)

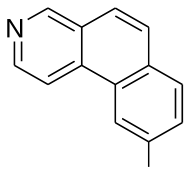

2e'

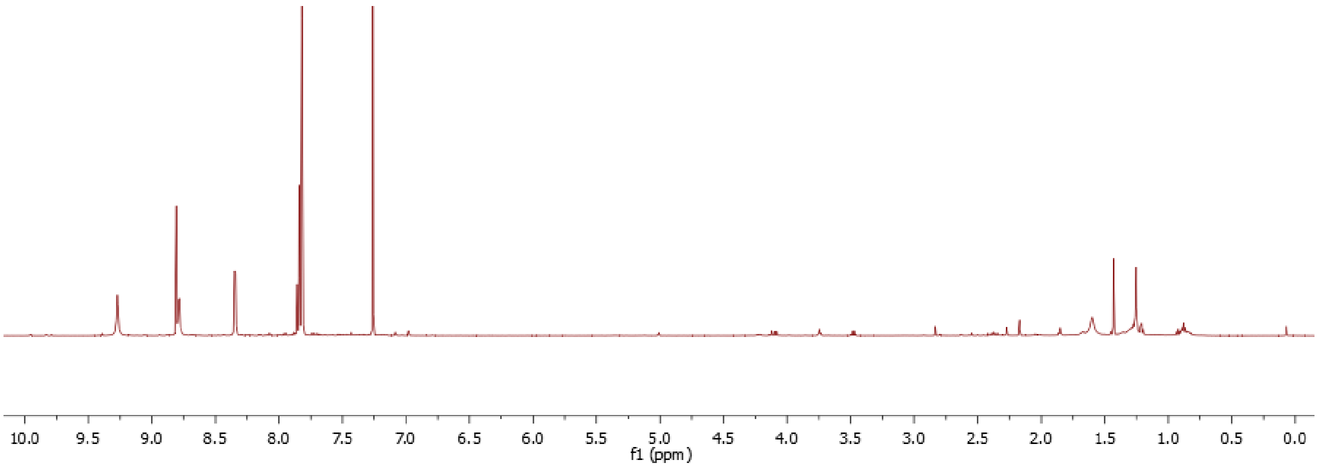

^{1}H NMR spectrum of 6-bromo-2-azaphenanthrene (2e')

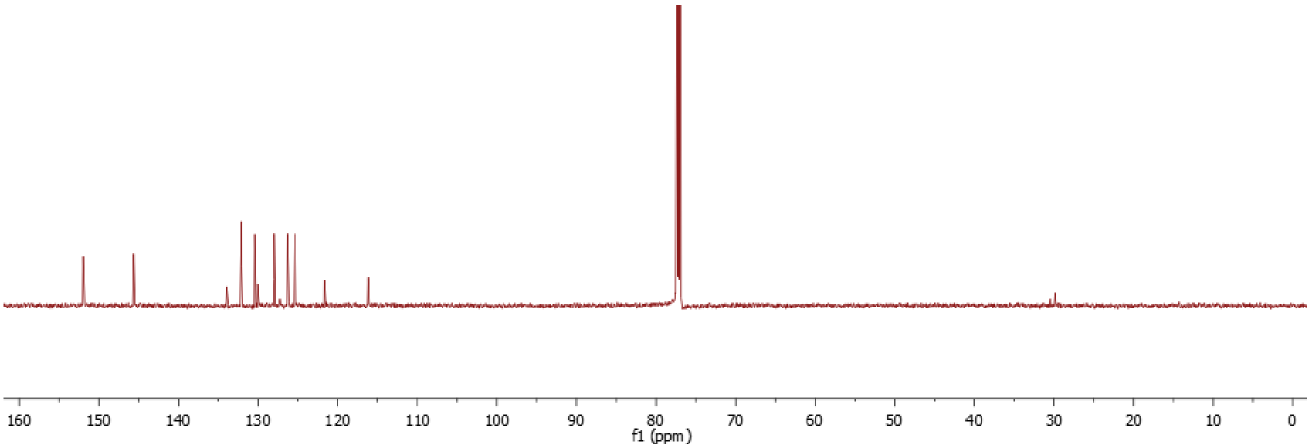

^{13}C NMR spectrum of 6-bromo-2-azaphenanthrene (2e')

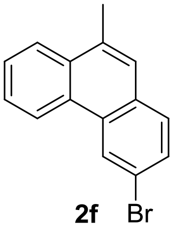

2f

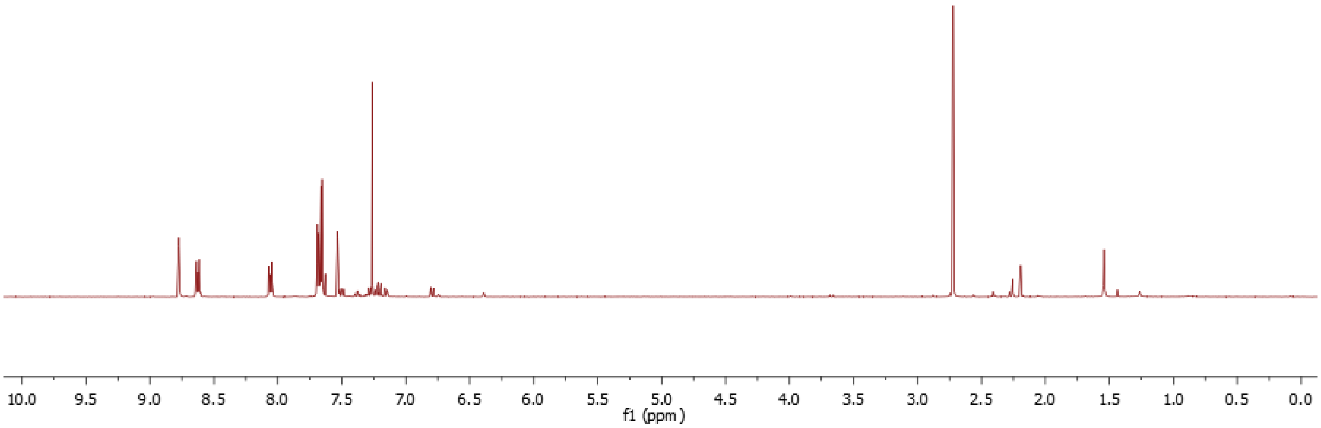

^{1}H NMR spectrum of 3-bromo-9-methylphenanthrene (2f)

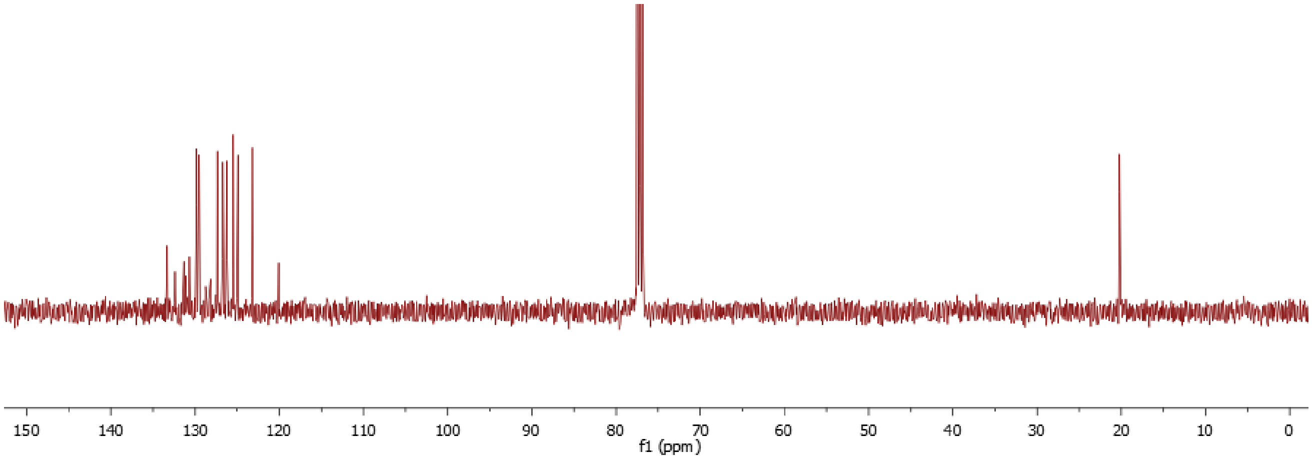

^{13}C NMR spectrum of 3-bromo-9-methylphenanthrene (2f)

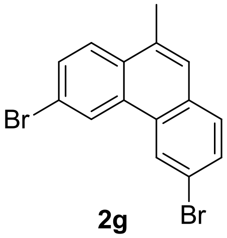

2g

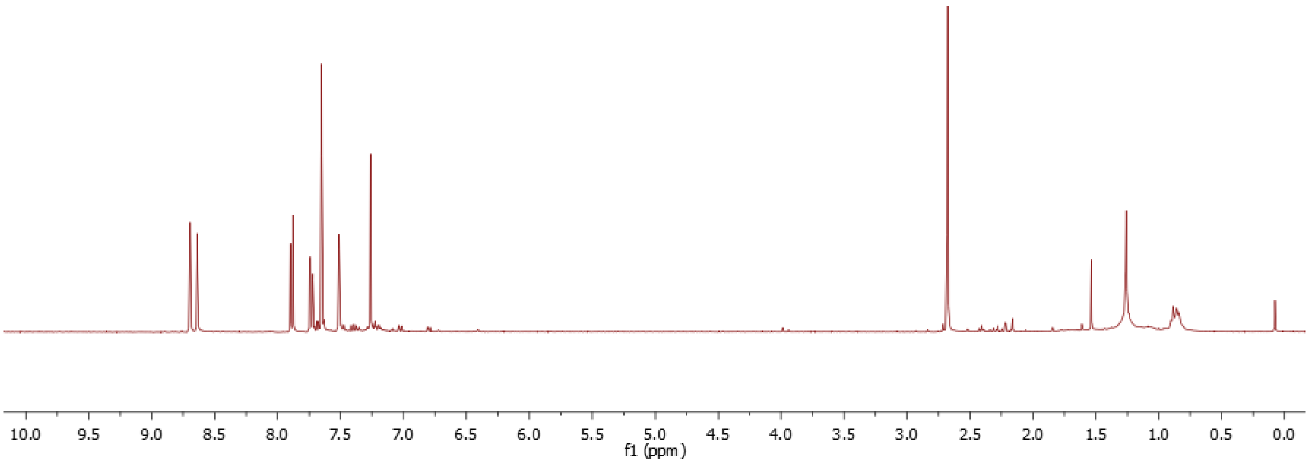

^{1}H NMR spectrum of 3,6-dibromo-9-methylphenanthrene (2g)

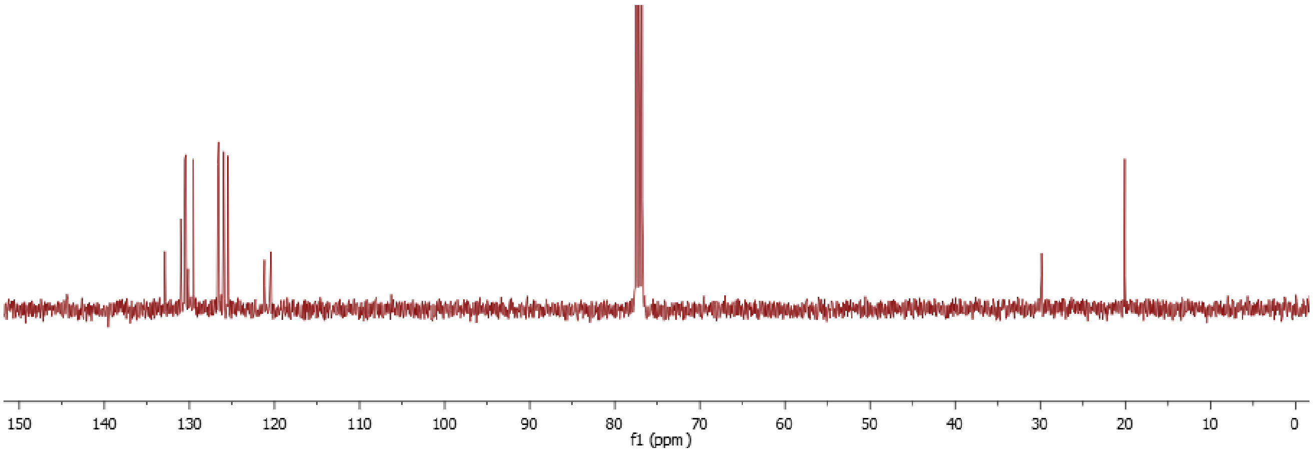

^{13}C NMR spectrum of 3,6-dibromo-9-methylphenanthrene (2g)

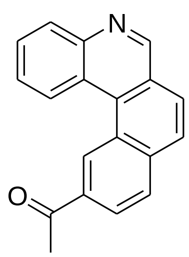

2k

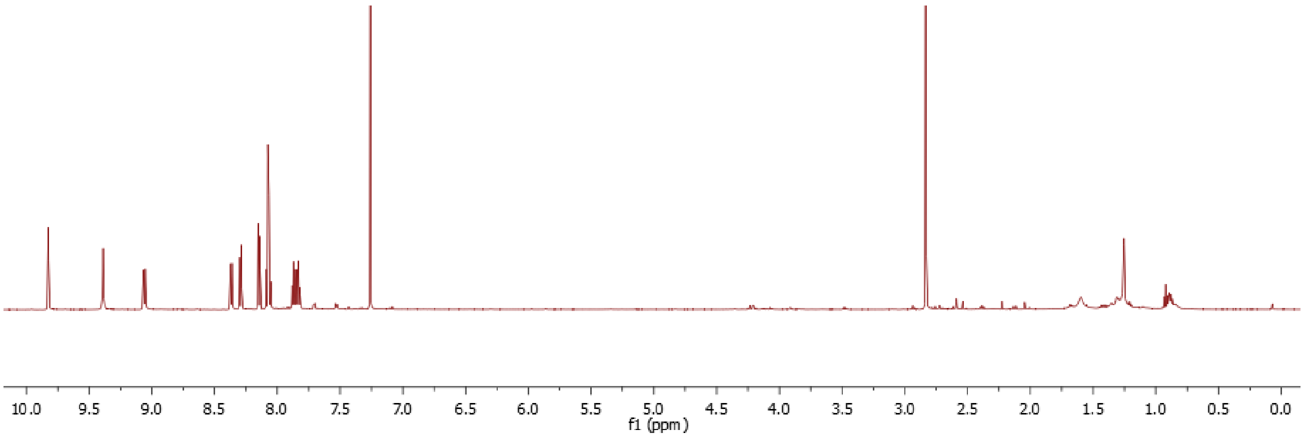

^{1}H NMR spectrum of 2-acetyl[4](8-azahelicene) (2k)

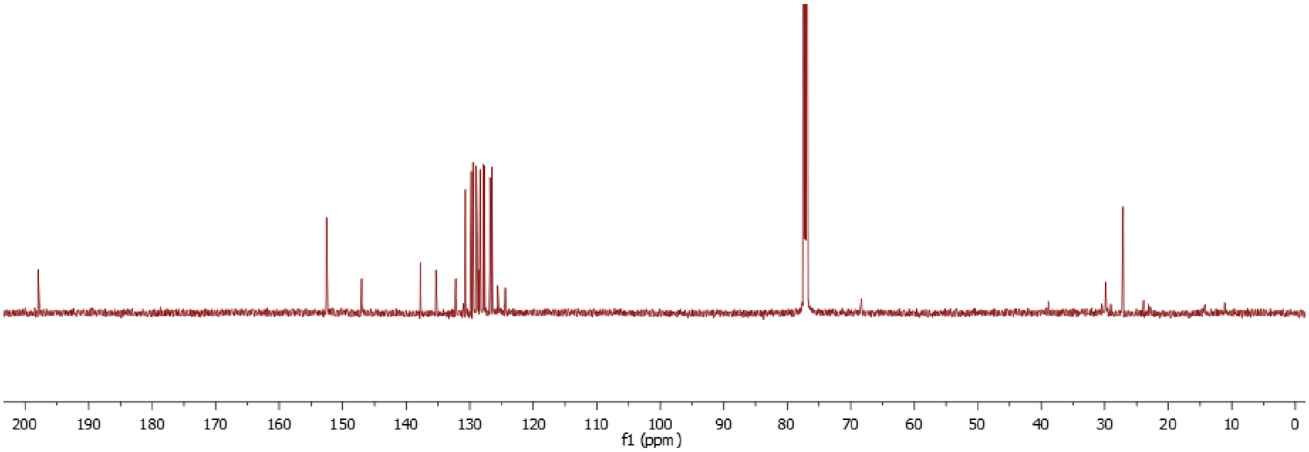

^{13}C NMR spectrum of 2-acetyl[4](8-azahelicene) (2k)

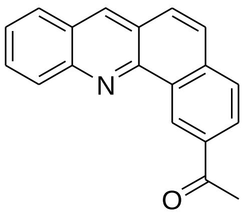

2k'

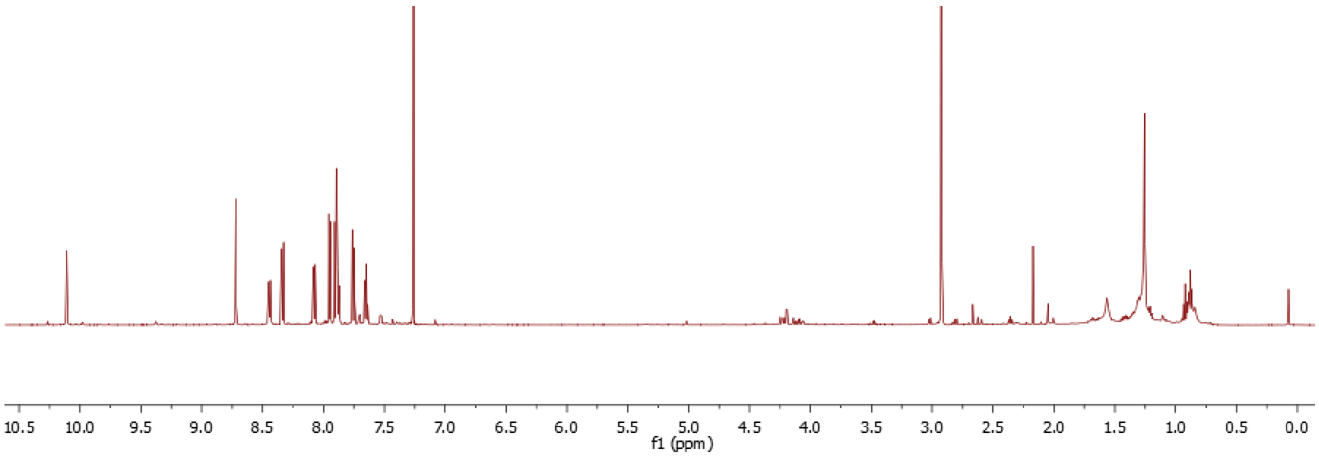

^{1}H NMR spectrum of 2-acetylbenz[*c*]acridine (2k')

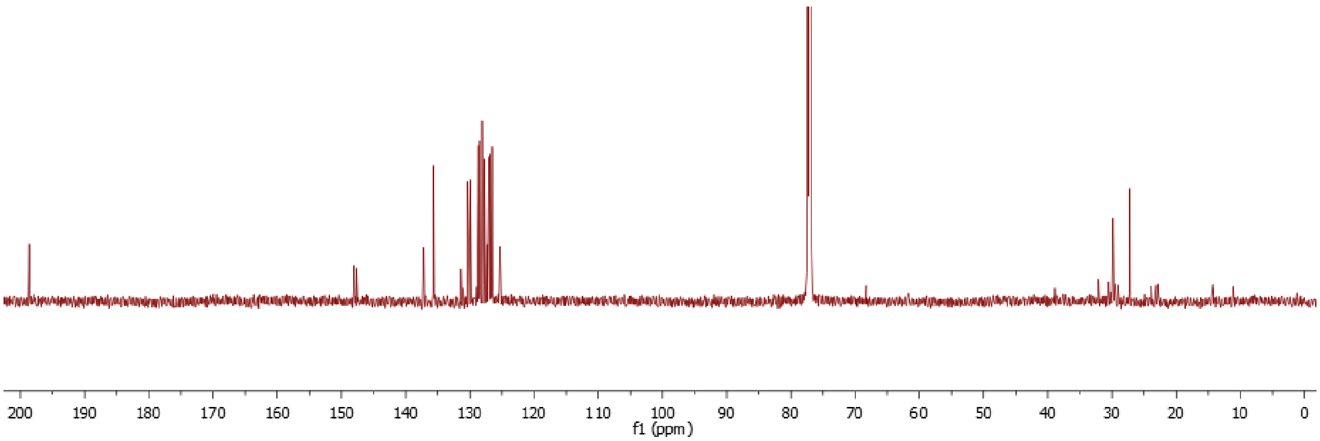

^{13}C NMR spectrum of 2-acetylbenz[*c*]acridine (2k')

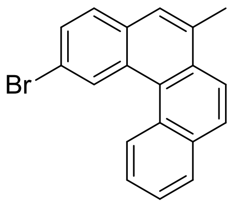

2l

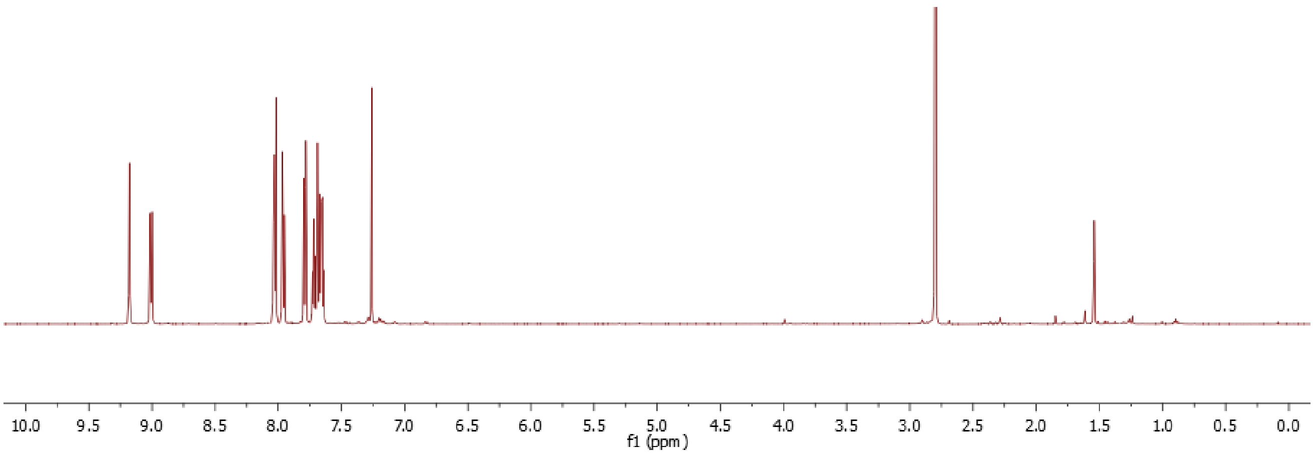

^{1}H NMR spectrum of 2-bromo-6-methyl[4]helicene (2l)

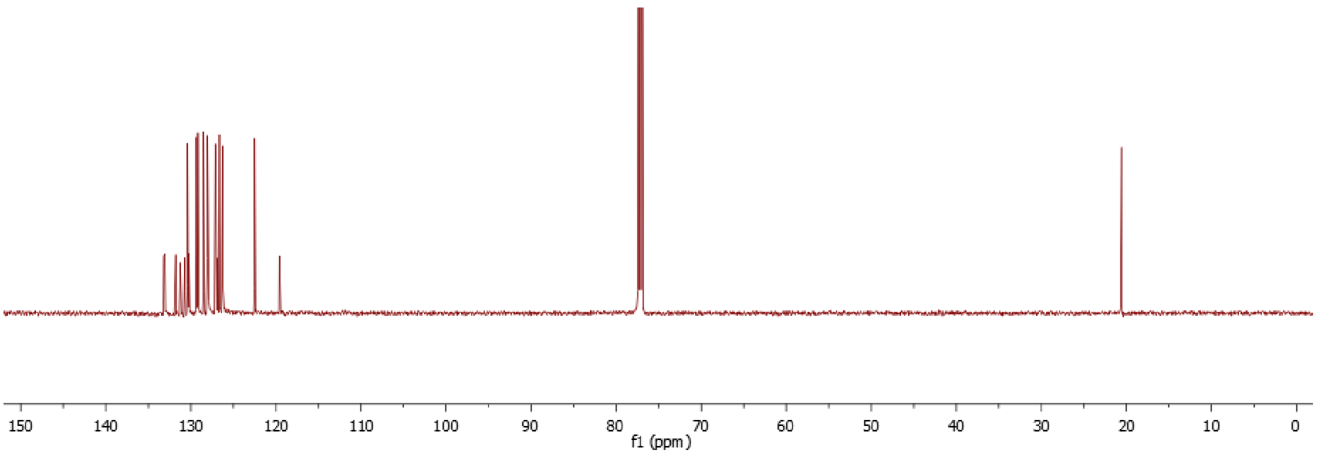

^{13}C NMR spectrum of 2-bromo-6-methyl[4]helicene (2l)

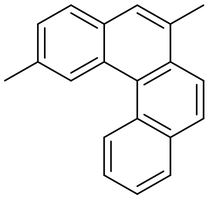

2m

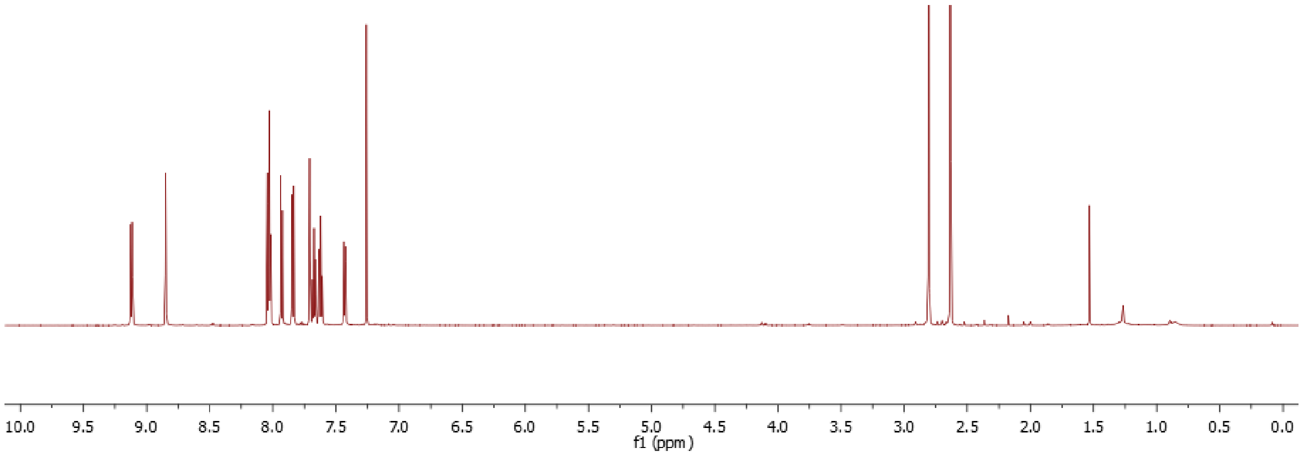

^{1}H NMR spectrum of 2,6-dimethyl[4]helicene (2m)

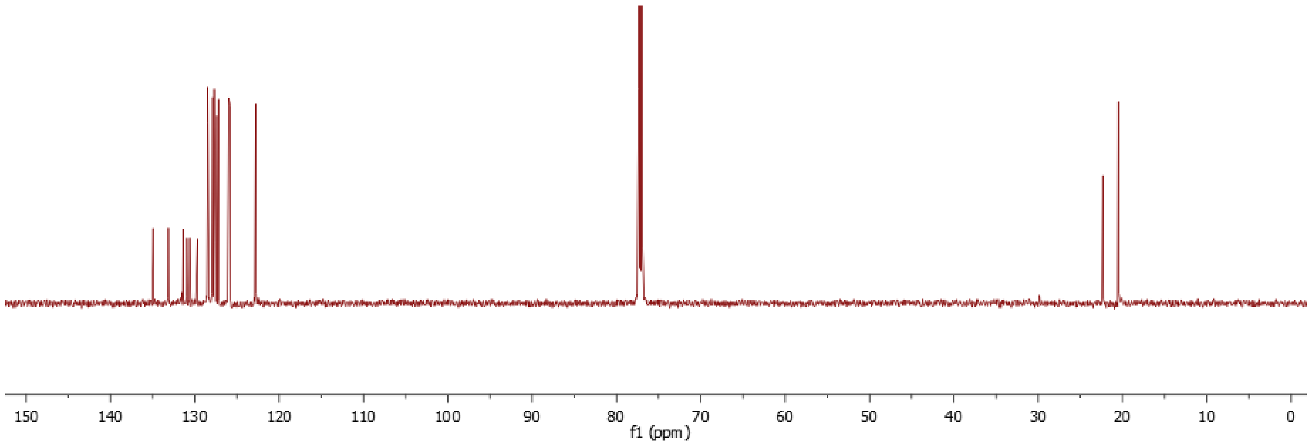

^{13}C NMR spectrum of 2,6-dimethyl[4]helicene (2m)

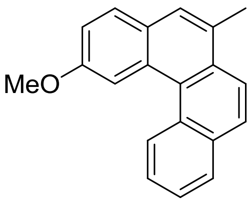

2n

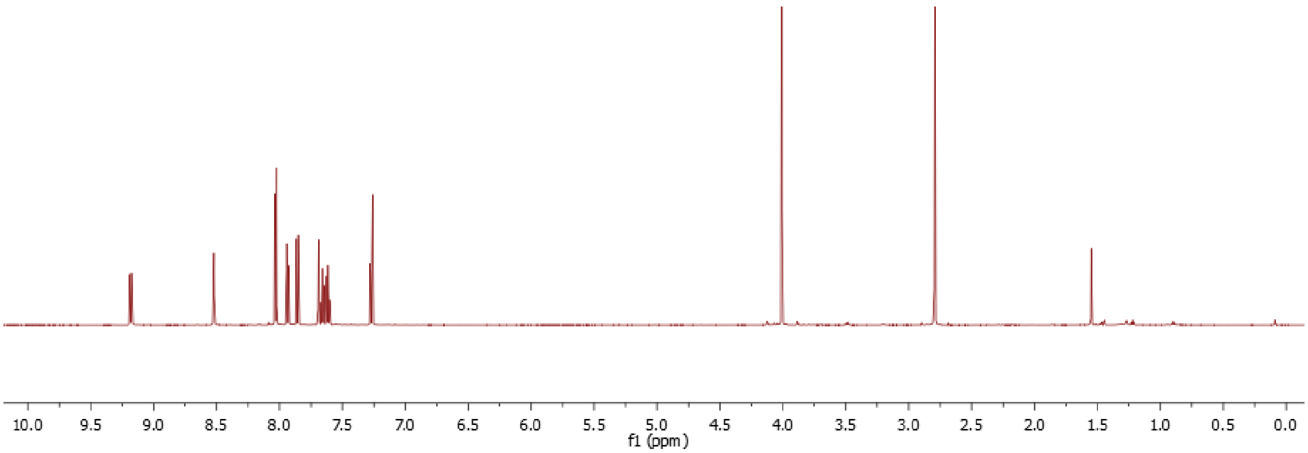

^{1}H NMR spectrum of 2-methoxy-6-methyl[4]helicene (2n)

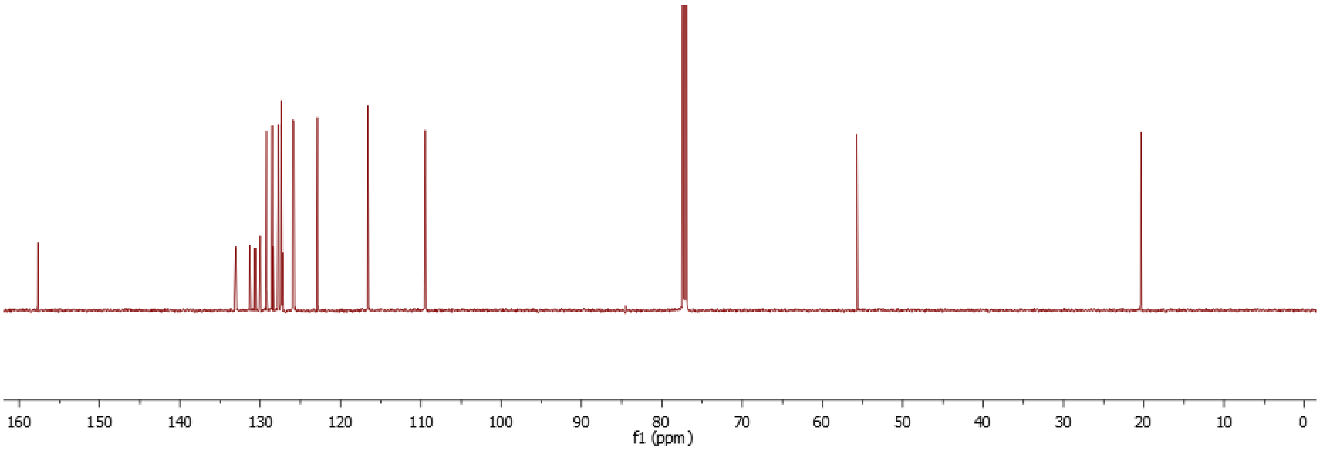

^{13}C NMR spectrum of 2-methoxy-6-methyl[4]helicene (2n)

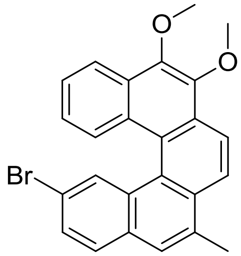

2o

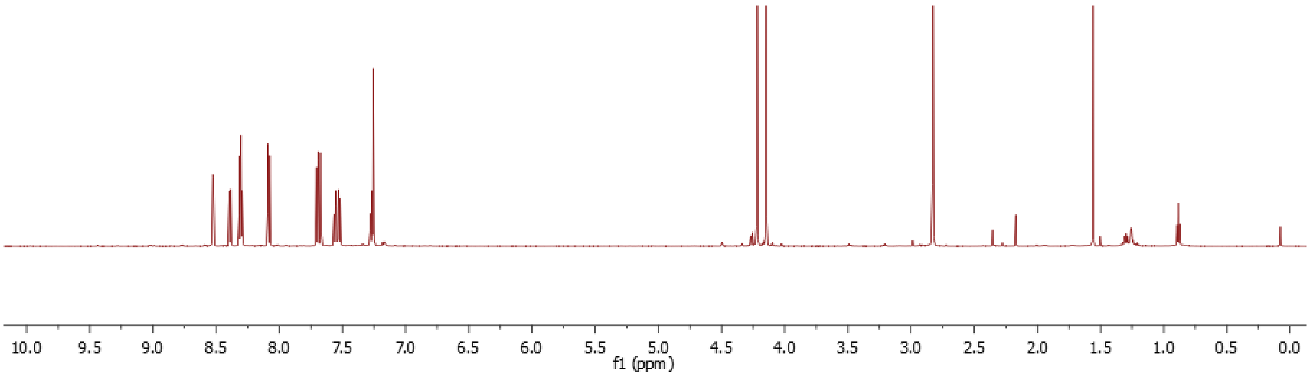

^{1}H NMR spectrum of 2-bromo-6-methyl-9,10-dimethoxy[5]helicene (2°)

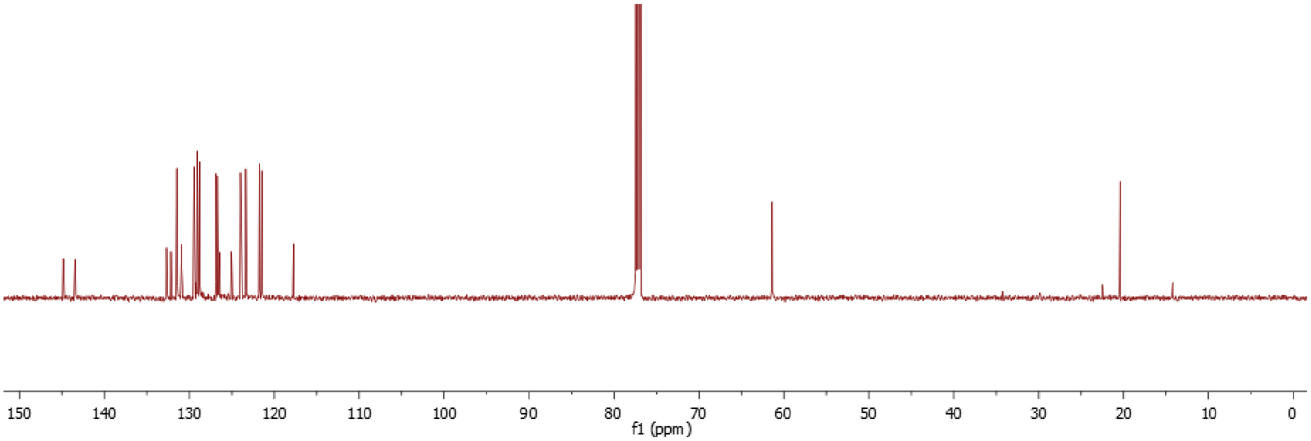

^{13}C NMR spectrum of 2-bromo-6-methyl-9,10-dimethoxy[5]helicene (2o)

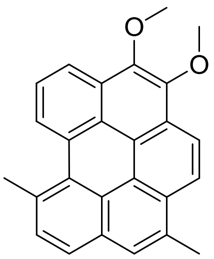

2p

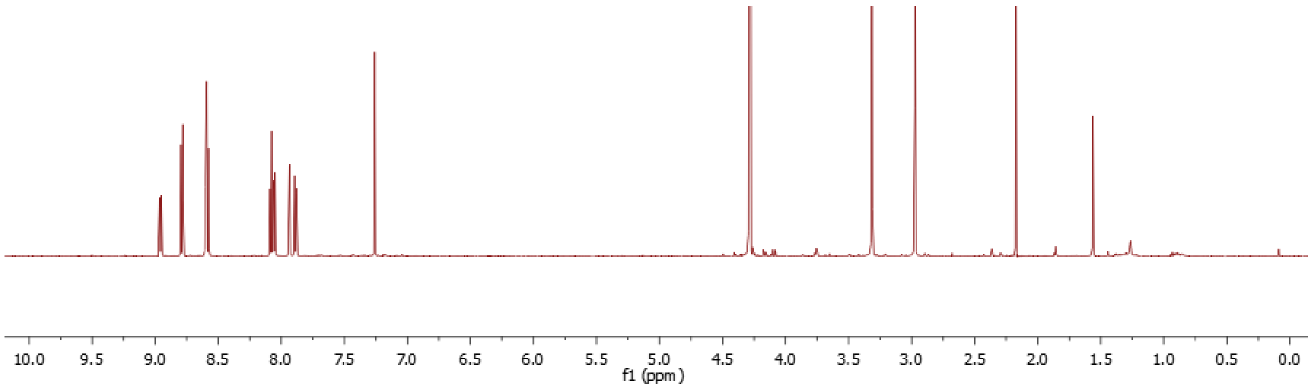

^{1}H NMR spectrum of 3,4-dimethoxy-8,12-dimethylbenzo[*ghi*]perylene (2p)

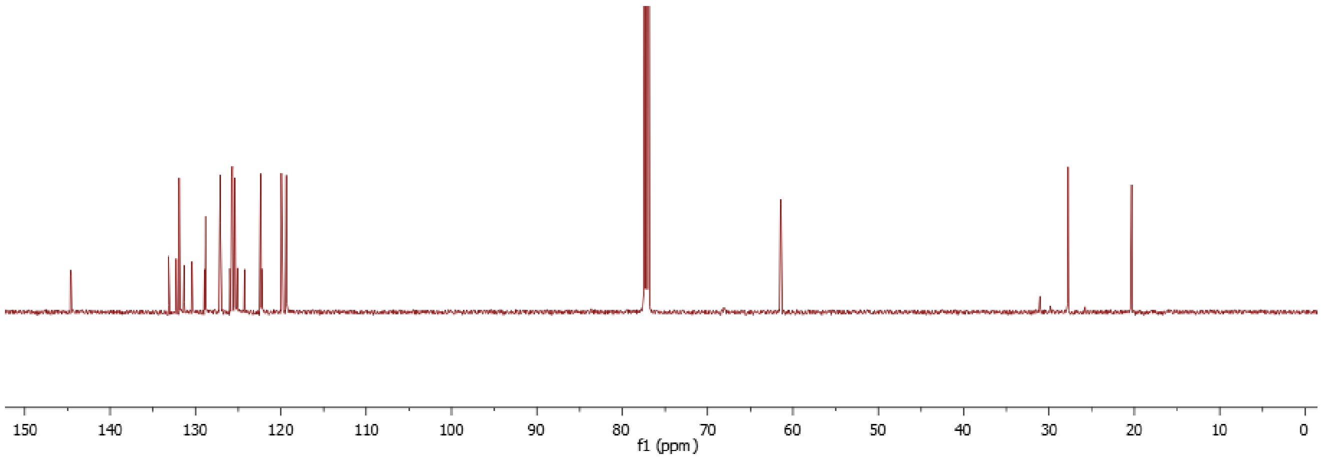

^{13}C NMR spectrum of 3,4-dimethoxy-8,12-dimethylbenzo[*ghi*]perylene (2p)

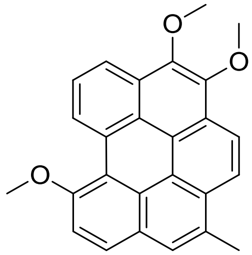

2q

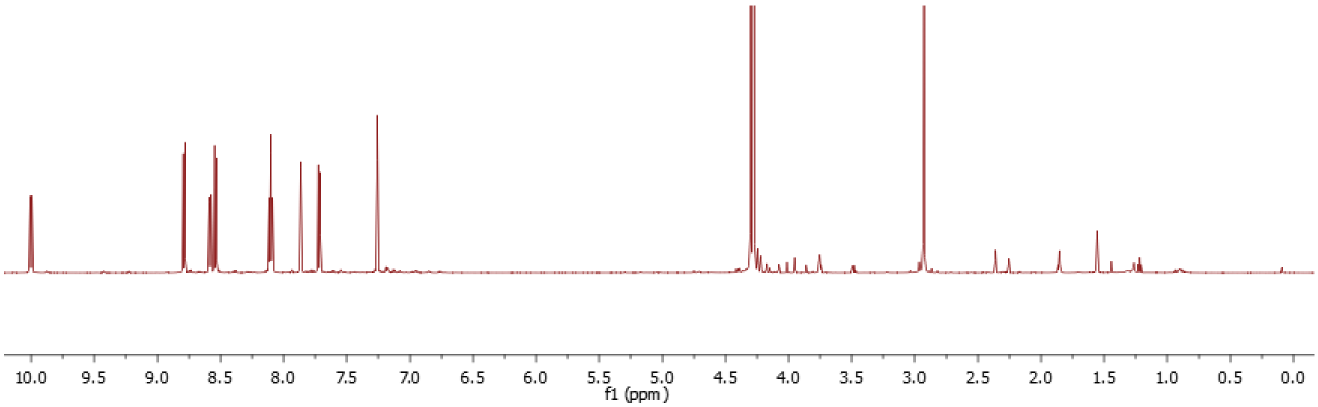

^{1}H NMR spectrum of 3,4,8-trimethoxy-12-methylbenzo[*ghi*]perylene (2q)

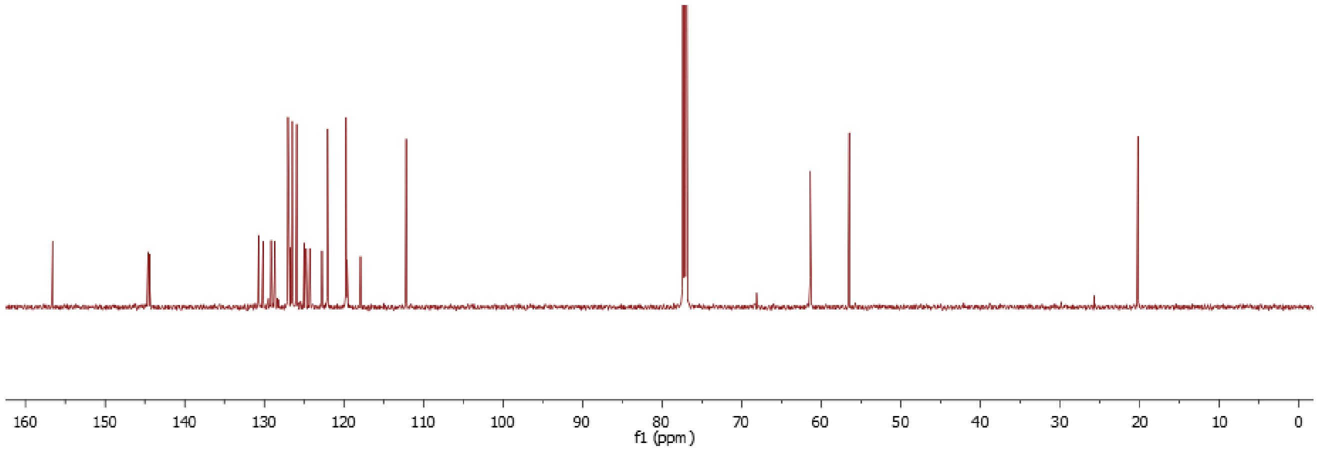

^{13}C NMR spectrum of 3,4,8-trimethoxy-12-methylbenzo[*ghi*]perylene (2q)

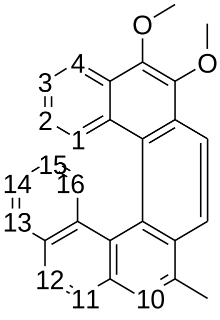

2r

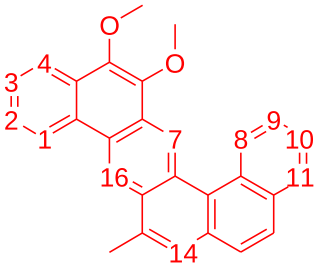

2r'

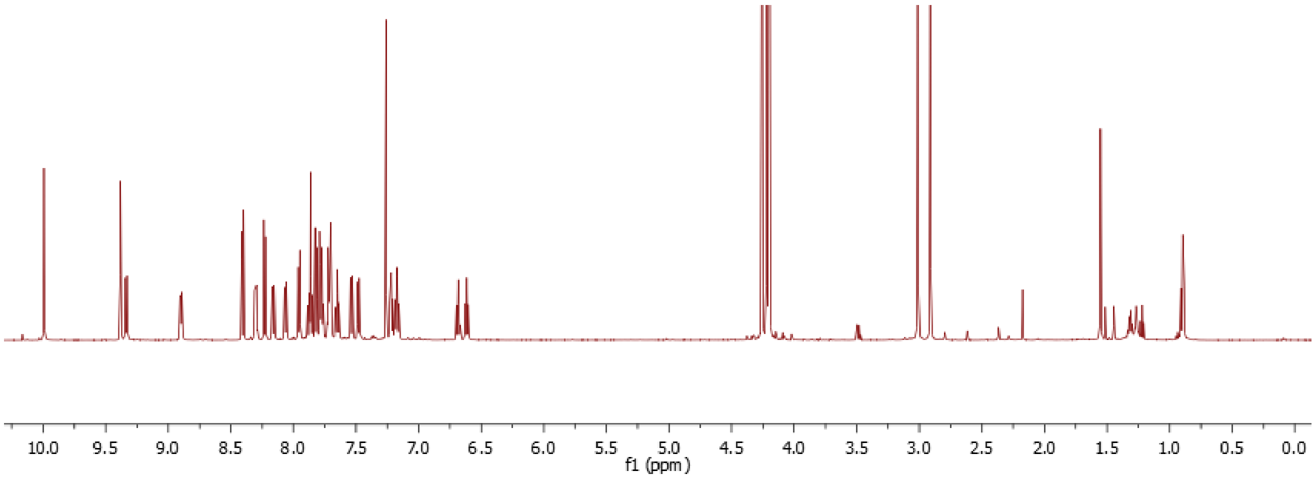

¹H NMR spectrum of the 1:1 mixture of 5,6-dimethoxy-9-methyl[6]helicene (2r) and its ribbon-like regioisomer 2r’

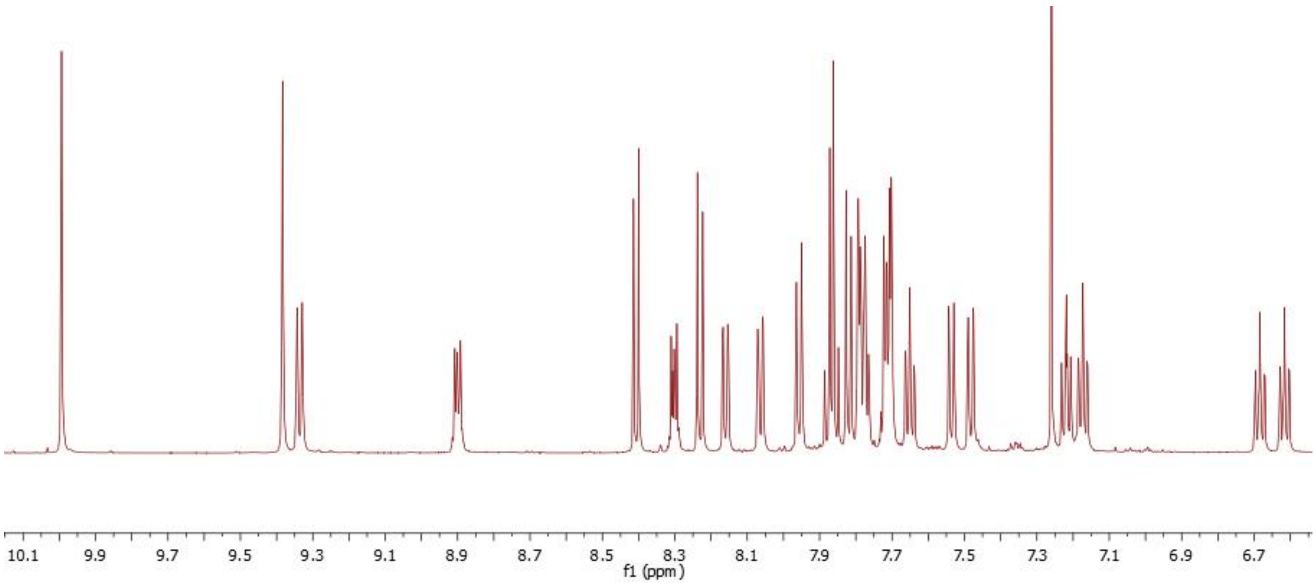

Expansion of the aromatic region of the ¹H NMR spectrum of the 1:1 mixture

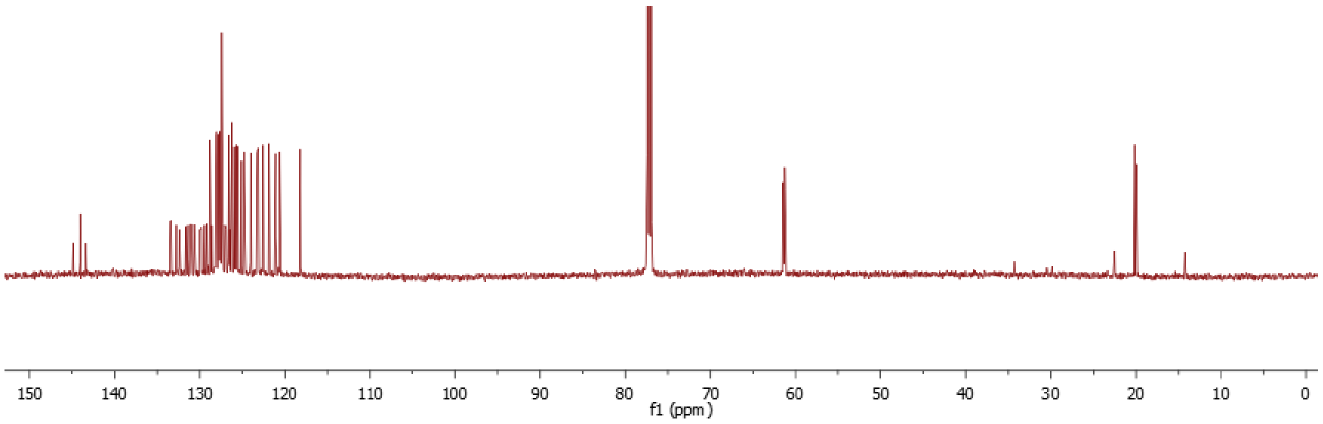

^{13}C NMR spectrum of the 1:1 mixture of 5,6-dimethoxy-9-methyl[6]helicene (2r) and its ribbon-like regioisomer 2r'
